# Genetic Evidence of Human Adaptation to a Cooked Diet

**DOI:** 10.1093/gbe/evw059

**Published:** 2016-03-15

**Authors:** Rachel N. Carmody, Michael Dannemann, Adrian W. Briggs, Birgit Nickel, Emily E. Groopman, Richard W. Wrangham, Janet Kelso

**Affiliations:** ^1^Department of Human Evolutionary Biology, Harvard University; ^2^Department of Evolutionary Genetics, Max Planck Institute for Evolutionary Anthropology, Leipzig, Germany; ^3^AbVitro Inc, Boston, Massachusetts; ^4^Columbia College of Physicians and Surgeons, New York, New York

**Keywords:** human evolution, food processing, metabolism, transcription

## Abstract

Humans have been argued to be biologically adapted to a cooked diet, but this hypothesis has not been tested at the molecular level. Here, we combine controlled feeding experiments in mice with comparative primate genomics to show that consumption of a cooked diet influences gene expression and that affected genes bear signals of positive selection in the human lineage. Liver gene expression profiles in mice fed standardized diets of meat or tuber were affected by food type and cooking, but not by caloric intake or consumer energy balance. Genes affected by cooking were highly correlated with genes known to be differentially expressed in liver between humans and other primates, and more genes in this overlap set show signals of positive selection in humans than would be expected by chance. Sequence changes in the genes under selection appear before the split between modern humans and two archaic human groups, Neandertals and Denisovans, supporting the idea that human adaptation to a cooked diet had begun by at least 275,000 years ago.

## Introduction

Ancestral humans underwent marked increases in body size and brain volume coupled with reductions in tooth and gut size beginning approximately 2 Ma (Aiello and Wheeler 1995). These biological features indicate the consumption of an easier-to-digest diet with increased caloric density, and have been argued to reflect a heavier reliance on animal foods (Aiello and Wheeler 1995; Stanford and Bunn 2001; Milton 2003; Speth 2010) and improved methods of food processing, including cooking (Wrangham et al. 1999; Wrangham and Carmody 2010). Cooking enhances nutrient digestibility and reduces diet-induced thermogenesis, thereby substantially increasing the energy gained from important hominin foods like meat and tubers (Carmody and Wrangham 2009; Carmody et al. 2011). Evidence that present-day humans cannot extract sufficient energy from uncooked wild diets, whether or not they include meat (Koebnick et al. 1999), has led to the suggestion that hunter-gatherers are biologically committed to these benefits of cooking (Wrangham and Conklin-Brittain 2003), including the provision of sufficient energy to fuel an exceptionally large brain (Fonseca-Azevedo and Herculano-Houzel 2012). The hypothesis that cooked food is obligatory for modern humans predicts genetic signals of human adaptation to a cooked diet. Indirect evidence of such adaptation—including pseudogenization of the masticatory myosin gene (*MYH16*) and of two bitter taste receptor genes (*TAS2R62* and *TAS2R64*) after the split from the common ancestor with chimpanzee, but prior to the split from the common ancestor with Neandertals and Denisovans (Perry et al. 2015)—encourages direct testing of this hypothesis.

Dietary modifications have previously been shown to cause genetic adaptation. Several populations with a legacy of dairying have acquired the ability to digest lactose into adulthood through persistence of the lactase enzyme (Bersaglieri et al. 2004; Gerbault et al. 2011), a trait that has evolved multiple times in the last approximately 7,000 years under strong positive selection (Tishkoff et al. 2007; Ranciaro et al. 2014). Additionally, populations with a history of consuming starch-rich foods have been argued to exhibit higher copy numbers of the gene encoding salivary amylase, the enzyme responsible for starch digestion in the mouth (Perry et al. 2007). The adoption of cooking is thought to be partly responsible for this adaptive covariation, as amylase is inefficient at digesting starch unless it has been first gelatinized by heat (Hardy et al. 2015). That diet-induced genetic adaptations exist among modern populations suggests that dietary modifications with longer evolutionary histories and broad systemic effects might produce more widespread genetic change.

Although the anatomical evidence from fossil *Homo* suggests that cooking began in the Lower Paleolithic, archaeological evidence for the control of fire is weak until the Middle Paleolithic (Gowlett and Wrangham 2013). Fire was certainly controlled by 250,000 years ago (James 1989), but is evidenced only occasionally back to 400,000 years; the oldest widely accepted date of anthropogenic fire is from Wonderwerk Cave, South Africa at 1 Ma (Berna et al. 2012). Although control of fire does not necessarily imply cooking, strong preferences for cooked items among great apes, combined with a readiness to wait for raw food to be cooked, suggest that cooking would likely have followed shortly thereafter (Wobber et al. 2008; Warneken and Rosati 2015). Notably, later putative dates for the origin of cooking overlap with the proposed split between modern humans and the last common ancestor of Neandertals and Denisovans, dated to between 275,000 and 765,000 years ago (Prufer et al. 2014), making it unclear whether cooking was present in the last common ancestor of our clade. Gelatinized starch granules embedded in the dental calculus of Neandertals suggest they were consuming cooked plant items by 50,000 years ago (Henry et al. 2011). However, sporadic evidence of fire use in cold-weather sites has led some to suggest that early Neandertals used fire opportunistically but did not control it (Roebroeks and Villa 2011; Sandgathe et al. 2011). Testing whether adaptation to a cooked diet occurred before or after the split between the modern human and Neandertal–Denisovan lineages could therefore help inform the timing of the control of fire.

Studies of genetic adaptation to a cooked diet cannot easily be performed in humans because of the rigorous experimental controls and tissue biopsies required (Somel et al. 2008). We therefore used gene expression changes in a model organism in response to raw and cooked diets to identify candidate genes that may have been affected by dietary change during human evolution. We then tested whether these genes exhibit expression differences between humans and nonhuman primates by comparing them to published genes showing human-specific expression patterns (Somel et al. 2008; Blekhman et al. 2010). We compared those genes affected by cooking and/or food type and then tested for signals of positive selection on these genes in humans. We focused on these effects in liver, a tissue for which diet has been shown to alter gene expression (Somel et al. 2008) and gene expression differences among humans and nonhuman primates have been catalogued (Blekhman et al. 2010).

## Materials and Methods

### Summary

In total, 24 adult male BALB/c mice were fed homogeneous diets of lean beef (*Bos taurus*) or sweet potato (*Ipomoea batatas*) served raw or cooked for a period of 5 days. On Day 5, mice were briefly fasted to encourage feeding on demand. Two hours into the Day 5 meal, mice were sacrificed and liver tissue was harvested and flash-frozen within 60 s of death. Total RNA was used to prepare barcoded cDNA libraries that were submitted for 75-bp paired-end sequencing on an Illumina HiSeq. After reads were filtered for quality and mapped to the mouse genome (NCBI37/mm9), differential expression was evaluated for the factors calorie intake (free-fed versus restricted), consumer energy balance (weight gain versus loss over 5 days of feeding), food type (meat versus tuber), and food preparation (raw versus cooked). Our data set of differentially expressed genes was compared with two external data sets (Somel et al. 2008; Blekhman et al. 2010) to interrogate its correlation with patterns of gene expression known to exist between humans and nonhuman primates. Differentially expressed genes that exhibited analogous patterns across primates were evaluated for enrichment in published lists of genes and promoter regions showing evidence of positive selection in the human lineage (Haygood et al. 2007; Kosiol et al. 2008). The timing of selective events was determined by comparison against a recent report of selection in the human lineage since the last common ancestor with Neandertals and Denisovans (Prufer et al. 2014), supplemented with a de novo analysis of selection based on nonsynonymous mutation rates in modern human populations. Full details on the subject animals, as well as the materials and protocols used in feeding trials, sample collection, library preparation, sequencing, and data analysis are provided below.

### Study Animals and Their Maintenance

Experiments were conducted in the Biological Research Infrastructure barrier facility under the supervision of the Harvard University Animal Care and Use Committee (Protocol 10-04). Inbred male BALB/c mice (*n* = 24, four sets of six littermates) were acquired from Charles River Laboratories at 21 days of age and cohoused with littermates under standard conditions (ad libitum chow and water; 12 h light/dark cycle; 22 ± 1 °C, 30**–**50% humidity) until growth rate tapered at 8 weeks of age. At 8 weeks of age, mice were housed individually in ventilated cages with a wire mesh floor to minimize coprophagy. To prevent contamination and loss of diet beneath the mesh floor, diets were administered in weighted Petri dishes with tops bearing four symmetrical feeding holes. Cages were sterilized daily, and fresh cotton nestlets and shacks were provided as enrichment. Mice acclimated to this experimental setup for 3 days before the start of diet manipulations.

### Experimental Diets

Diets consisted of organic lean beef eye round roast (*B. taurus*) or organic sweet potato tubers (*I. batatas*) served either raw, cooked, or cooked but in a restricted ration that allowed us to evaluate the effects of a cooked diet given negative energy status. We used domestic species grown in the eastern United States that could be supplied without freezing, which is known to affect material and nutritive properties in both meat (Ballin and Lametsch 2008) and tubers (Mondy and Chandra 1979). Foods were sourced fresh daily (meat: Savenor’s Market, Cambridge, MA; tubers: Broadway Market, Cambridge, MA). For the raw meat diet (MRF [meat/raw/free-fed]), meat was sliced into standard cuboids (3.0 × 1.5 × 1.3 cm^3^; 10.0 ± 0.2 g) and weighed into unlimited rations (20.0 ± 0.3 g). For cooked meat diets (MCF [meat/cooked/free-fed], MCR [meat/cooked/restricted]), standard raw meat cuboids were first weighed into free-fed (MCF; 20.0 ± 0.3 g) or restricted (MCR; 10.0 ± 0.3 g) rations and placed into Pyrex Petri dishes of known mass. Rations were roasted in their dishes in batches of six dishes at 200 °C for 12 min. Cooking time was determined to result in internal temperatures of 65–70 °C, the temperature at which collagen gelatinizes (Purslow 2005), equivalent to medium-well done. Cooked samples were allowed to cool for 15 min, and were then weighed in their dishes to assess cooked weight. For the raw tuber diet (TRF [tuber/raw/free-fed]), tubers were cut into standard cuboids (3.0 × 1.8 × 1.3 cm^3^; 10.0 ± 0.2 g) and weighed into unlimited rations (40.0 ± 0.5 g). For cooked tuber diets (TCF [tuber/cooked/free-fed], TCR [tuber/cooked/restricted]), raw tuber cuboids were portioned into free-fed (TCF; 40.0 ± 0.5 g) or restricted (TCR; 20.0 ± 0.3 g) rations, arranged in foil packets of uniform batch size (four pieces, 40.0 ± 0.5 g), and roasted in a convection oven at 204 °C for 25 min. Cooking time was determined empirically to produce complete gelatinization of starch by polarized light microscopy. After cooking, tuber diets were allowed to cool for 15 min, and were then transferred into preweighed Petri dishes and weighed a second time to determine cooked weight. Once prepared, all diets were sealed with parafilm to prevent further evaporation prior to feeding. All diets were prepared under sterile conditions and fed at room temperature within 3 h of preparation. Technical replicates of the MRF, MCF, MCR, TRF, TCF, and TCR diets, prepared from the same starting materials and by the same methods as the diets fed to mice, were analyzed for energy and macronutrient content at the Nutritional Ecology Laboratory at Harvard University using standard biochemical assays (see supplementary table S1, Supplementary Material online). The measured energy contents per gram of these technical replicates were later combined with data on food intake to give the absolute caloric intake per mouse per day. In addition, technical replicates of MRF and MCF diets from five different preparation batches were swabbed and cultured according to published protocols (Smith et al. 2015) in order to rule out contamination by the common meat-associated pathogenic bacterial taxa *Escherichia coli* and *Staphylococcus*.

### Feeding Protocol

Mice were reared for 5 days on MRF, MCF, MCR, TRF, TCF, or TCR diets (*n* = 4 per diet), with littermates divided symmetrically across diet groups. Diets were presented at the same time each day to give a standardized data collection cycle. During this daily intervention, mice were weighed during a period of inactivity. Food refusals from the past 24 h were collected, weighed to monitor fresh weight intake, and later freeze-dried to determine dry weight intake.

### Tissue Harvest

At the end of the feeding trial (day 5), mice were fasted overnight (12 h) to promote consumption of food on demand. Two hours before sacrifice, mice were presented with their assigned diets and in all cases began eating immediately. Body mass was taken immediately prior to euthanization via CO_2_ inhalation. Duplicate 50-mm^2^ sections of the right lobe of the liver were excised within 60 s of death using sterile, RNase-free instruments. The tissues were flash frozen in liquid nitrogen and stored at −80 °C until analysis.

### Library Preparation, Sequencing, and Base Calling

Total RNA was extracted from liver samples using the RNeasy Mini Kit (Qiagen, Valencia, CA). Tissue disruption was achieved by grinding under liquid nitrogen using a sterile, RNase-free mortar and pestle. RNA yield and integrity were quantified by Agilent bioanalyzer, and all samples met our inclusion criteria of RNA ≥8 μg with RIN ≥8.0. The Illumina RNAseq sample preparation protocol and kit (RS-100-0801) as well as the Illumina Paired End library preparation protocol and kit (PE-102-1001) were used for library preparation. Briefly, poly-A transcripts were enriched from the total RNA using poly-T-coated magnetic beads. The poly-A RNA was fragmented using an Ambion buffer (70 °C, 5 min). RNA fragments were reverse transcribed into cDNA using random priming (Invitrogen SuperScript II). Second-strand synthesis was performed in the same reaction using RNaseH and DNA polymerase I. Fragments were blunt-ended using T4 DNA polymerase (50 overhang fill-in) and Klenow DNA polymerase (30–50 exonuclease activity). During this process a deoxyadenosine was added to the 3′-end of the DNA fragments and T4 DNA ligase was then used to ligate forked adapters. Library clean-up was carried out using SPRI beads. Fragments were then amplified with overhanging primers that extended the adapters to the final length required for sequencing.

We sequenced our libraries on four lanes of an Illumina HiSeq 2500 following vendor protocols, obtaining 75-bp paired-end reads. HiSeq sequencing runs were analyzed starting from raw intensities. Base calling and quality score calculation were performed using the freeIBIS base caller (Renaud et al. 2013), trained on ϕX174 control reads. Reads with more than five bases below a base quality score of 10 were excluded. Adapter sequences were trimmed and all remaining reads were kept for downstream analysis.

### Mapping and Expression

After excluding two lanes due to sequencing artifacts we mapped all remaining reads to the mouse genome (NCBI37/mm9) using the TopHat mapper (Trapnell et al. 2009) and standard parameters. We were able to map 97.4% (range across individuals: 94.9–99.6%) of all reads. All mapped reads above a minimum mapping quality of 30 were kept and assigned to genes defined by the mouse ENSEMBL gene annotation (version 65). All genes with a read count greater than zero in at least two individuals were defined as expressed.

### Differential Expression Analysis

We used the multifactor model provided by the DESeq package (Anders and Huber 2010) to test for differential expression for the factors food type (meat versus tuber), food preparation (raw versus cooked), caloric intake (free-fed versus restricted), and consumer energy balance (weight gain versus loss over 5 days of feeding). Genes with an adjusted *P* value <0.05 were defined to be differentially expressed. The *P*-value correction was performed based on the method of Benjamini–Hochberg (Benjamini and Hochberg 1995).

### Effect of Consumer Energy Balance on Gene Expression

Since weight gain and loss were highly correlated with food type and food preparation, we developed a test to determine whether consumer energy balance (weight gain vs. loss over 5 days of feeding) had an effect on gene expression that was independent of food type or food preparation. We focused on mice fed the cooked tuber diet because some individuals fed this diet gained weight whereas others lost weight. We used the multifactor model provided by the DESeq package (Anders and Huber 2010) to test for differential expression between mice gaining weight and mice losing weight. Genes with an adjusted *P* value <0.05 were defined to be differentially expressed. We found no gene with false discovery rate (FDR) < 0.05, suggesting that consumer energy balance had little effect on differential gene expression in this experiment.

### Functional Enrichment Analysis

We tested differentially expressed genes for functional enrichment in the gene ontology (GO) (Gene Ontology Consortium 2000) and the Kyoto Encyclopedia of Genes and Genomes (KEGG) pathway (Kanehisa and Goto 2000) databases. Differentially expressed genes were tested for enrichment by comparing them with all expressed genes using a hypergeometric test. For the GO, we used the FUNC package (Prufer et al. 2007) and defined functional categories to be significantly overrepresented if the corresponding family-wise error rate (FWER) was smaller than 0.05. Overrepresentation of differentially expressed genes in a KEGG pathway was computed by running the GOstats R package (Falcon and Gentleman 2007), and pathways with a *P* value <0.05 were considered significantly enriched (see supplementary data S1, Supplementary Material online).

### External Data Sets

#### Mouse Microarray Data Set

We used microarray expression data from an experiment by Somel et al. (2008), in which liver transcription was compared between mice fed human diets versus chimpanzee diets (ArrayExpress accession numbers GSE6285 and GSE6297). We computed expression estimates for all individuals using the R affy and gcrma packages (Wu et al. 2004), excluding all individuals fed the pellet diet.

#### Primate RNAseq Data Set

We used RNAseq-based liver transcription data from multiple human, chimpanzee, and rhesus macaque individuals provided by Blekhman et al. (2010) (ArrayExpress series accession number GSE17274). We mapped reads to the corresponding genomes hg19, pantro2, and rhemac2 using TopHat (Trapnell et al. 2009) with standard parameters and all mapped reads with a minimum mapping quality of 30 were retained. We obtained expression values for each individual by running cufflinks (Trapnell et al. 2010) on all mapped reads. Gene annotations for human genes were obtained from Ensembl (version 59) and orthologous regions in chimpanzee and rhesus macaque were defined using liftover (Hinrichs et al. 2006). Only exons that were identified by liftover in all three species were used.

### Correspondence with External Data Sets

We correlated expression differences for candidate genes in our study with expression differences in the mouse microarray and primate RNAseq data sets described above. In order to correlate expression changes, we matched the factors in our study to those of the other experiments (mouse microarray [Somel et al. 2008]: Human cafeteria/McDonald’s diet versus chimpanzee diet; primate RNAseq [Blekhman et al. 2010]: Human versus chimpanzee/rhesus macaque), matching our “meat” and “cooked” treatments to human subjects and our “tuber” and “raw” treatments to nonhuman primate subjects. To simplify the assignment, only one factor (either food type or food preparation) was considered at a time. When comparing food type, meat (whether raw or cooked) was assigned to humans and tuber (whether raw or cooked) was assigned to nonhuman primates. When comparing food preparation, cooked foods (whether meat or tuber) were assigned to humans and raw foods (whether meat or tuber) were assigned to nonhuman primates. We computed the percentage of genes with the same fold change in the compared experiments. We then tested whether the percentage of fold change agreement was significantly higher for the differentially expressed genes compared with all other expressed genes using the one-sided Fisher’s exact test. In the case of the primate data set, we only considered genes that showed human-specific expression, that is, a gene is consistently either upregulated or downregulated in humans compared with both chimpanzees and rhesus macaques. The *P*-value correction was performed based on the method of Benjamini–Hochberg (Benjamini and Hochberg 1995).

### Transcription Factor Analysis

Using a set of transcription factors and their predicted target genes (Matys et al. 2006) we explored whether expression changes in target genes tended to agree between our mouse expression data and the Blekhman primate expression data (Blekhman et al. 2010). As before, we matched “meat” and “cooked” treatments to human subjects and “tuber” and “raw” treatments to nonhuman primate subjects. We then tested whether the overlap was greater than expected by chance using the one-sided Fisher's exact test, comparing the overlap of target genes for particular transcription factors to the overlap for all remaining genes.

### Overlap with Positively Selected Genes

Using a likelihood ratio test on the phylogeny of species trees to assign changes to different mammalian lineages (Kosiol et al. 2008) identified positively selected genes for multiple mammalian species including primates. We used the positively selected genes reported by Kosiol and colleagues for both the human and the chimpanzee lineages, and defined all genes to be positively selected that showed a *P* value <0.05 in the corresponding likelihood ratio test. For all factors in our study and their corresponding differentially expressed genes, we computed the overlap with the positively selected genes in both species and compared the fraction of overlapping genes to the overlap of all remaining expressed genes using the one-sided Fisher’s exact test with the alternative hypothesis that we find a greater proportion of genes overlapping than expected. The *P*-value correction was performed based on the method of Benjamini–Hochberg (Benjamini and Hochberg 1995).

### Overlap with Recently Positively Selected Genes

Using a hidden Markov model (Prufer et al. 2014) detected long genomic regions where the high-coverage genomes of the Neandertal and Denisovan fall outside the variation seen in modern humans. When comparing the regions identified using the Neandertal with those identified using the Denisovan, the top 200 regions were found to overlap significantly more than expected, and genes in these regions were therefore proposed as candidates for having undergone recent positive selection on the human lineage. As for the overlap with positively selected genes on the human lineage since chimpanzee, for each factor we compared the overlap of differentially expressed genes with the overlap of not differentially expressed genes using the one-sided Fisher’s exact test with the alternative hypothesis that we find a greater proportion of genes overlapping than expected. The *P*-value correction was performed based on the method of Benjamini–Hochberg (Benjamini and Hochberg 1995).

### Overlap with Positively Selected Promoter Regions

We obtained positively selected promoter regions from a study by Haygood et al. (2007). To identify these regions, Haygood et al. (2007) used a likelihood ratio rest to compare evolutionary rates between the promoter regions and nearby intronic regions of genes. They identified genes with regulatory regions putatively under positive selection on the human lineage. We overlapped differentially expressed genes in our study with genes that showed evidence of positive selection in their promoter region (*P* value < 0.05). As for the overlap with positively selected genes in the human lineage since chimpanzee, for each factor we compared the overlap of differentially expressed genes with the overlap of not differentially expressed genes using the one-sided Fisher’s exact test with the alternative hypothesis that we find a greater proportion of genes overlapping than expected. The *P*-value correction was performed based on the method of Benjamini–Hochberg (Benjamini and Hochberg 1995).

## Results

### Cooking Increases Food Energy Value

**Fig. 1.—:**
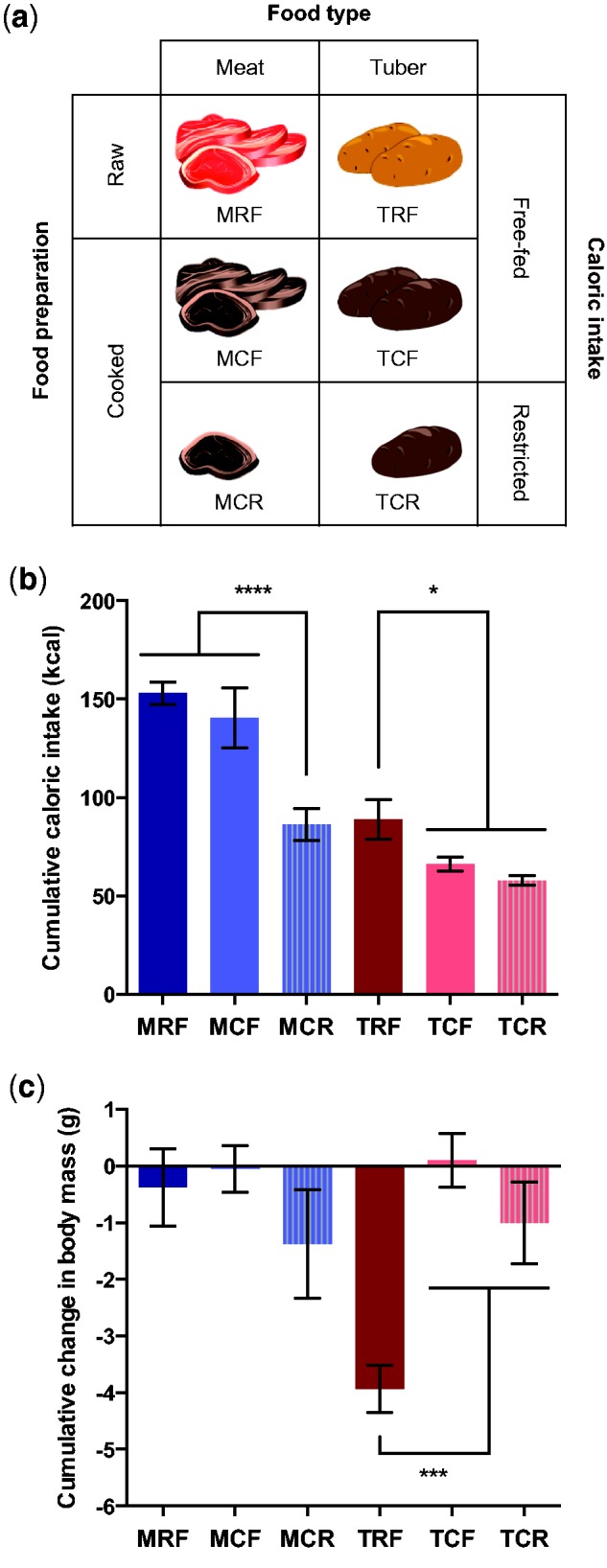
Summary of the study design. (*a*) Mapping of diet treatments to the key factors tested in this study: Food type (meat versus tuber), food preparation (raw versus cooked), and caloric intake (free-fed versus restricted). (*b*) Cumulative caloric intake over the 5-day feeding trial by diet treatment (one-way ANOVA; *F* = 83.54; *P* < 0.0001). (*c*) Cumulative change in body mass over the 5-day feeding trial by diet treatment (one-way ANOVA; *F* = 17.10; *P* < 0.0001). Data are reported as mean ± SEM, with significant post hoc differences among treatments indicated by asterisks (Tukey’s HSD; ****P < 0.0001; ***P < 0.001; *P < 0.05). See also supplementary table S1, Supplementary Material online.

We reared 24 adult male BALB/c mice for 5 days on diets of beef or sweet potato fed raw or cooked (supplementary table S1, Supplementary Material online). Cooked diets were presented in free-fed or restricted rations to generate variation in caloric intake and consumer energy balance (fig. 1*a*). As predicted, ration restriction led to lower caloric intake versus free-fed diets of the same preparation (two-way ANOVA; restriction: *F* = 49.70; *P* < 0.0001; food type: *F* = 133.6; *P* < 0.0001; interaction: *F* = 26.83; *P* = 0.0002) and ultimately to weight loss (two-way ANOVA; restriction: *F* = 12.88; *P* = 0.0037; food type: *F* = 0.6038; *P* = NS; interaction: *F* = 0.1109; *P* = NS). Interestingly, although free-fed cooked diets were associated with lower caloric intake than free-fed raw diets (two-way ANOVA; food preparation: *F* = 12.38; *P* = 0.0048; food type: *F* = 188.7; *P* < 0.0001; interaction: *F* = 1.019; *P* = NS; fig. 1*b*), the cooked diets nevertheless allowed for weight retention whereas raw diets led to weight loss (two-way ANOVA; food preparation: *F* = 65.51; *P* < 0.0001; food type: *F* = 40.06; *P* < 0.0001; interaction: *F* = 47.43; *P* < 0.0001; fig. 1*c*), replicating our prior finding that cooking increased net energy gain per gram of these foods (Carmody et al. 2011).

### Food Type and Food Preparation Impact Liver Gene Expression

**Fig. 2.—:**
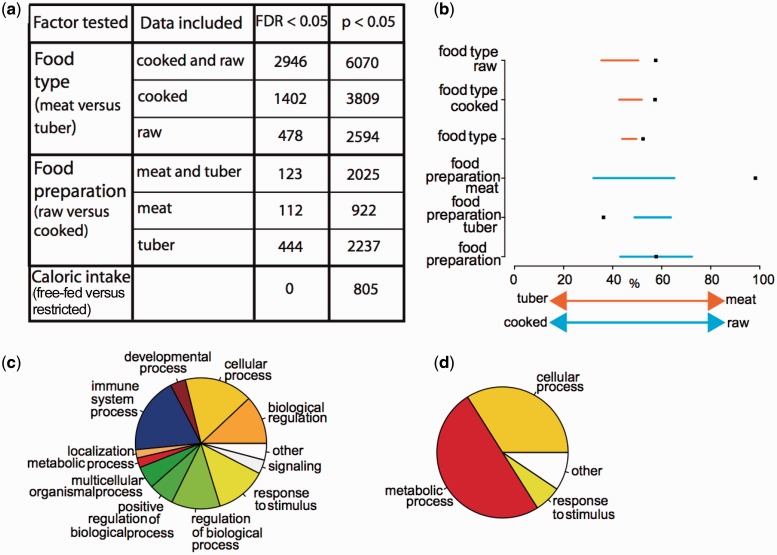
Summary of changes in gene expression. (*a*) Number of differentially expressed genes by food type, food preparation, and caloric intake given an FDR <0.05 (corrected for multiple comparisons) or *P* value <0.05 (not corrected for multiple comparisons). The first column reflects the factor tested (i.e., “food type” evaluates differences between meat and tuber samples), whereas the second column reflects the data set(s) included in the test. The number of differentially expressed genes for combined data sets (e.g., “raw and cooked”) may exceed the sum of the underlying data sets (e.g., “raw” + “cooked”) due to enhanced model power. (*b*) For genes that were differentially expressed by food type or food preparation, the percentages (*x* axis) of genes that show an increased expression in meat (for food type) and raw (for food preparation) diets are shown as black squares, and the corresponding 95% CI for the random expectation in orange (food type; *P* < 0.001) or blue (food preparation; *P* < 0.001). (*c*, *d*) Classification of functionally enriched categories in the GO (Gene Ontology Consortium 2000) for all differentially expressed genes (*P* value < 0.05) between (*c*) raw versus cooked diets, and (*d*) meat versus tuber diets. See also supplementary figures S1–S3 and table S2, Supplementary Material online.

Sequencing of the RNA extracted from liver tissue harvested 2 h after the start of a meal revealed that neither caloric intake (free-fed versus restricted) nor consumer energy balance (weight gain versus loss over 5 days of feeding) resulted in significant differences in gene expression. By contrast, differences in food type (meat versus tuber) and food preparation (raw versus cooked) accounted for large numbers of differentially expressed genes (fig. 2*a*).

Expression differences by food type corresponded to underlying differences in the macronutrient contents of meat versus tuber (supplementary fig. S1, Supplementary Material online). Genes associated with carbohydrate metabolism were more highly expressed in mice fed tuber (84.8 ± 0.9% carbohydrate, dry-weight basis) compared with mice fed meat (carbohydrate content below the limit of detection) regardless of cooking, consistent with the richer carbohydrate contents of tuber diets. In addition, these genes were more highly expressed on raw compared with cooked tuber diets, suggesting that increased physiological investment was necessary to digest raw plant material (Carmody and Wrangham 2009). Supporting this idea, we observed higher expression of *Amy1* (ENSMUSG00000074264), the gene coding for salivary amylase, in mice fed raw tuber versus raw meat (*P* < 0.05, FDR > 0.05), and in mice fed raw tuber versus cooked tuber (*P* < 0.05, FDR > 0.05). Such data are consistent with previous arguments that higher expression of *AMY1* reflects a higher demand for starch digestion (Perry et al. 2007; Axelsson et al. 2013; Hardy et al. 2015). Conversely, genes involved in lipid metabolic processes were highly expressed in mice fed meat (11.5±0.6% lipid, dry-weight basis) compared with mice fed tuber (0.8±0.2%). We also observed lower expression of genes related to lipid metabolism in cooked compared with raw meat, a result potentially explained by lipid loss during cooking and/or by lower digestive requirements for cooked lipids (Wrangham and Carmody, forthcoming).

Notably, 110 of the 112 (98%) genes that were differentially expressed between mice fed raw versus cooked meat showed higher expression in mice on the raw meat diet, a larger bias than for any other set of differentially expressed genes (fig. 2*b* and supplementary table S2, Supplementary Material online). Functional enrichment analysis revealed these genes to be highly enriched (FWER <0.05) for immune-related functions (fig. 2*c* and *d*, supplementary fig. S2 and data S1, Supplementary Material online). Cultures of *E. coli* and *Staphylococcus* prepared from swabs of the raw and cooked meat diets (Smith et al. 2015) offered no evidence of contamination by these common foodborne pathogenic taxa, suggesting that immune upregulation on the raw meat diet was not simply due to high pathogen load.

### Expression Differences Associated with Meat and Cooking Mirror Those Observed between Humans and Nonhuman Primates

**Fig. 3.—:**
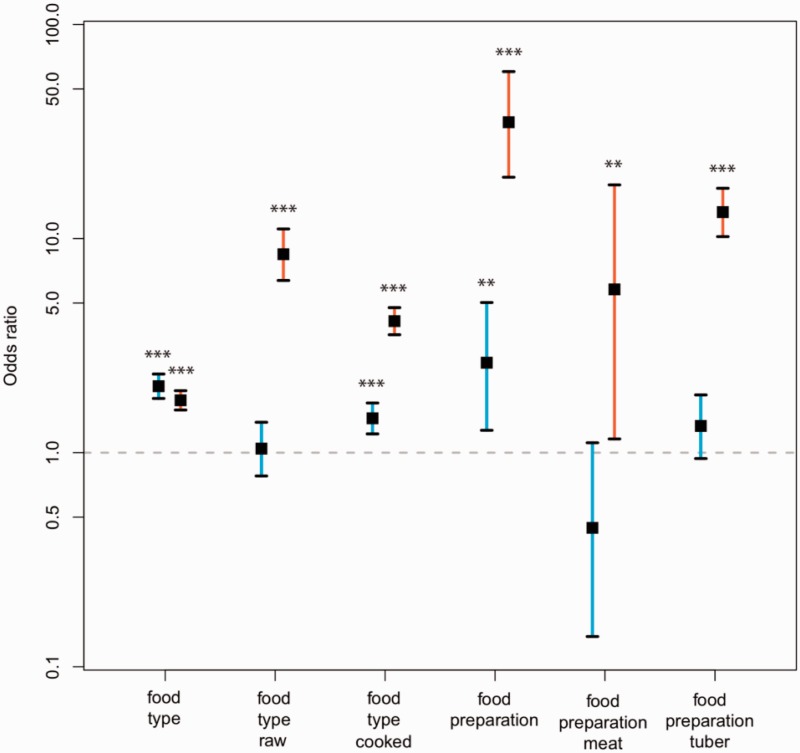
Correspondence with external gene expression data sets. We compared the expression differences observed in our study with the expression differences observed in two previously published studies reporting expression differences between humans and nonhuman primates. We matched our “meat” and “cooked” treatments to human subjects and our “tuber” and “raw” treatments to nonhuman primate subjects. Specifically, we compared the genes that Somel et al. (2008) reported as being differentially expressed between mice fed human versus chimpanzee diets with the genes in our study that were differentially expressed between meat versus tuber (food type), respectively, and cooked versus raw (food preparation), respectively. Similarly, we compared the genes that Blekhman et al. (2010) reported as being differentially expressed between humans and nonhuman primates with the genes in our study that were differentially expressed between meat versus tuber (food type), respectively, and cooked versus raw (food preparation), respectively. We computed the odds ratio (black squares ± 95% CI) by comparing the proportion of differentially expressed genes in our study that show the same expression changes observed by Somel et al. (2008) (orange) or Blekhman et al. (2010) (blue) against the proportion for genes that were not differentially expressed. Odds ratios exceeding chance are indicated by asterisks (***P < 0.001; **P < 0.01). See also supplementary table S3, Supplementary Material online.

When increased meat consumption and cooking are treated as human-associated dietary traits, the genetic signatures of food type and food preparation that we observed mirror differences previously reported to exist among mice fed human versus chimpanzee diets (Somel et al. 2008) and among human versus nonhuman primate livers (Blekhman et al. 2010). Our mice fed meat exhibited liver gene expression patterns that were more similar to mice fed a human diet, and more similar to human liver, than was the case for our mice fed tuber (fig. 3 and supplementary table S3, Supplementary Material online). Similarly, expression in our mice fed cooked food more closely resembled that of mice fed a human diet and that of human liver than did expression in our mice fed raw food. By contrast, our mice fed tuber or raw foods showed liver expression patterns more similar to those of mice fed a chimpanzee diet and to expression patterns observed among nonhuman primates. We postulated that differences in liver gene expression might be partially governed by changes in the activity of specific transcription factors. However, after correction for multiple testing we found no significant enrichment in the expression of genes targeted by any particular transcription factor for any comparison.

### Putative Cooking-Related Genes Show Evidence of Positive Selection in the Human Lineage

**Fig. 4.—:**
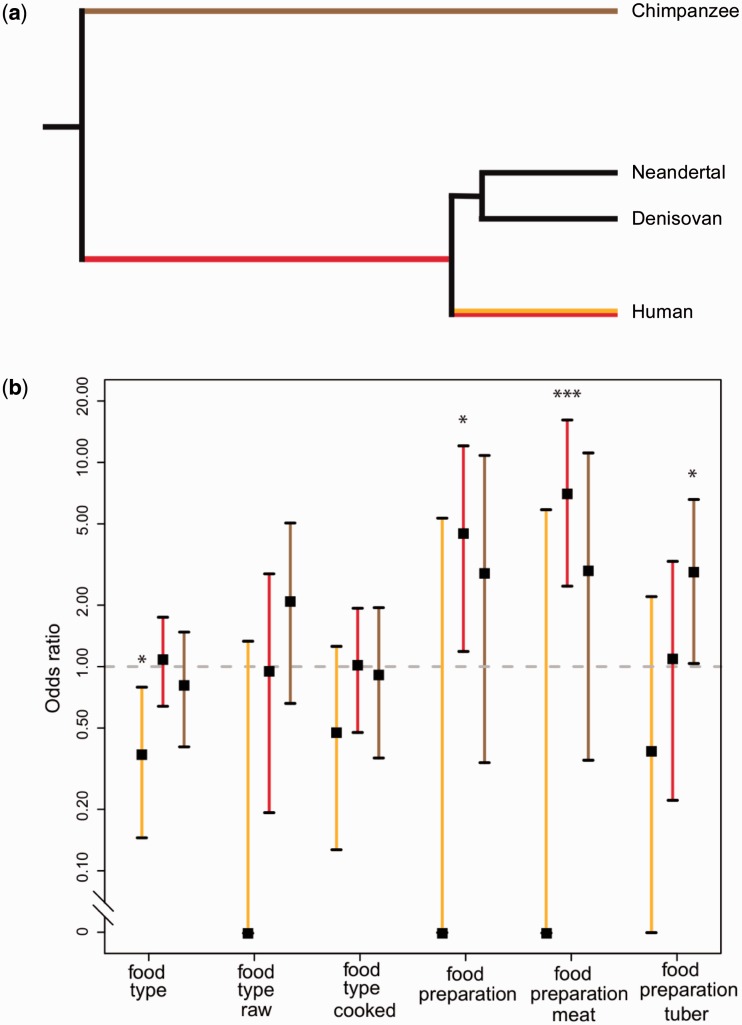
Positive selection on cooking-related genes in the human lineage. (*a*) Reference phylogeny of chimpanzees, Neandertals, Denisovans, and modern humans: brown = chimpanzee lineage, red = human lineage since the split from chimpanzees, yellow = modern human lineage since the split from Neandertals and Denisovans. (*b*) Enrichment of differentially expressed genes within sets of genes bearing evidence of positive selection in the human lineage since the split from Neandertals and Denisovans (Prufer et al. 2014), since the split from chimpanzees (Kosiol et al. 2008), and in the chimpanzee lineage (Kosiol et al. 2008). We computed the odds ratio (black squares ±95% CI) by comparing the proportion of differentially expressed genes that bear evidence of positive selection against the proportion for genes that were not differentially expressed. Odds ratios exceeding chance are indicated by asterisks (***P < 0.001; *P < 0.05). See also supplementary tables S4 and S5, Supplementary Material online.

Differences in gene expression between humans and chimpanzees could reflect consumer experience or hard-wiring of physiology by natural selection. To test whether genes affected by food type and food preparation might have been targets of selection during human evolution (fig. 4*a*), we investigated whether genes that were differentially expressed by diet in mice were enriched among genes with evidence of positive selection in the human lineage (Kosiol et al. 2008). No significant enrichments were observed for genes associated with food type (supplementary table S4*a*, Supplementary Material online). By contrast, we found that genes associated with food preparation exhibited more overlap than expected by chance, particularly in the comparison of raw versus cooked meat (fig. 4*b* and supplementary table S4*a*, Supplementary Material online). A total of seven putatively selected cooking-related genes were identified (table 1), of which six are involved in immune processes. Importantly, the overlap between genes differentially expressed in mice and those positively selected on the chimpanzee lineage was not significant, suggesting that the putative selective events were restricted to the human lineage. Promoter regions under positive selection on the human lineage have previously been shown to be enriched for nutrition-related functions (Haygood et al. 2007). We therefore examined whether cooking-related genes in our data set showed a greater than expected overlap with positively selected promoters, but found enrichment for neither food type nor food preparation (supplementary table S4*b*, Supplementary Material online).

**Table 1:** Putative Cooking-Related Genes

Gene ID/ENSEMBL ID Mouse	Gene ID/ENSEMBL ID Human	Differential Expression	
		Raw versus Cooked Food	Raw versus Cooked Meat
Marco/ENSMUSG00000026390	MARCO/ENSG00000019169	X	X
Lilra6/ENSMUSG00000030427	LILRA5/ENSG00000187116	X	X
Lilrb3/ENSMUSG00000058818	LILRA5/ENSG00000187116	X	X
Dusp4/ENSMUSG00000031530	DUSP4/ENSG00000120875	X	
Lilra5/ENSMUSG00000070873	LILRA5/ENSG00000187116		X
Gm14548/ENSMUSG00000074417	LILRA5/ENSG00000187116		X
Tnfrsf11a/ENSMUSG00000026321	RANK/ENSG00000141655		X

Note.—Set of positively selected genes that were differentially expressed in raw versus cooked food and in raw versus cooked meat.

Additionally, because the benefits of cooking could in theory reduce the selection on some genes to maintain function, we also investigated whether cooking-related genes might have lost their functional constraint in the human lineage. We identified two human pseudogene families for which the functional equivalents in mice exhibited lower expression on cooked diets (supplementary fig. S3, Supplementary Material online), including the major urinary protein (*Mup*) genes, as well as CMP-sialic acid hydroxylase (*Cmah*), which is the enzyme responsible for Neu5Gc production. Additional studies of selection on these and other genes with strong cooking-related differential expression could enable a pathway-level assessment of the mechanisms by which cooking influences the biological value of food, a fundamental topic that remains unexplored.

### Positive Selection in Putative Cooking-Related Genes Predates the Origin of Modern Humans

We used high-coverage genome sequences from two archaic hominins, a Neandertal and a Denisovan, both from Denisova cave in the Altai mountains (Meyer et al. 2012; Prufer et al. 2014), to determine whether it is more likely that selection on adaptations to a cooked diet occurred before or after the split between the human and Neandertal–Denisovan lineages. First, we compared the putatively selected cooking-related genes in our data set against a list of genes with evidence of recent selection on the human lineage since the split with Neandertals and Denisovans (Prufer et al. 2014), but found no overlap exceeding chance (fig. 4*b* and supplementary table S5, Supplementary Material online). Second, we identified nonsynonymous single nucleotide changes (SNCs) that are fixed in modern human populations (1000 Genomes Project Consortium 2012) and asked whether these pre- or postdate the split of humans from Neandertals and Denisovans. Among putatively selected cooking-related genes, all observed nucleotide changes occurred before the split (raw versus cooked foods: 4 genes with 6 SNCs; raw versus cooked meat: 6 genes with 11 SNCs; table 1). However since 98.7% of the 23,819 SNCs observed in all genes expressed in our data set occurred before the split, we cannot exclude the possibility that this distribution occurs by chance. Nevertheless, both analyses suggest that if genes associated with cooking have undergone selection, the selective events likely occurred at least 275,000–765,000 years ago (Prufer et al. 2014). In addition, we note that the *Mup* and *Cmah* genes that show a lower expression in mice fed cooked food are pseudogenized not only in modern humans but also in the Neandertal and Denisovan genomes, providing an additional line of evidence that changes associated with cooking are likely to predate the split between the modern and archaic lineages.

## Discussion

All human societies cook. This practice distinguishes us from other species and has been argued to be obligatory given our biological commitment to a high-quality diet and the fact that cooking substantially increases net energy gain (Wrangham and Conklin-Brittain 2003; Carmody and Wrangham 2009; Wrangham and Carmody 2010). However our current understanding of human digestive specialization compared with other primates is largely restricted to anatomical rather than physiological features, including diminution of mouth, teeth, stomach, and large intestine. Although these changes strongly indicate adaptation to reliance on easily chewed and rapidly digested food, some raw foods fit this description, for example, fruits, marrow, brains, liver, honey, and select items like seeds that benefit substantially from nonthermal processing. Without understanding molecular adaptations to a cooked diet, it is therefore impossible to be sure whether habitual cooking has shaped our physiology, and if so, how.

In this study, we provide the first evidence that eating cooked versus raw foods influences liver gene expression. We also find abundant differences in gene expression between diets of meat versus tuber, with enrichment of lipid-related metabolic processes on meat diets and carbohydrate-metabolic processes on tuber diets. By contrast, manipulating conditions of caloric intake or consumer energy balance had minimal impact on gene expression. Together, these results suggest that differential expression was driven primarily by changes in nutrient availability and/or specific physiological processes, as opposed to simply energy flux.

Genes differentially expressed between mice fed raw and cooked meat were almost exclusively upregulated on the raw meat diet. These genes were highly enriched for immune-related functions, supporting the common but poorly tested assumption that cooking of meat prevents a costly immune response (Ragir 2000; Carmody and Wrangham 2009). However, the specific triggers of immune upregulation on the raw meat diet remain unclear. Cultures of two common pathogenic taxa prepared from the raw versus cooked meat diets did not suggest contamination, although differences in the activity of other foodborne pathogens cannot be ruled out from the available data. Interestingly, meat consumption has been shown to trigger inflammation in humans due to the formation of antibodies against *N*-glycolylneuraminic acid (*Neu5Gc*), a monosaccharide lost from human cell surfaces due to a human-specific inactivating deletion (Chou et al. 1998). However, wild-type mice produce endogenous *Neu5Gc* (Chandrasekharan et al. 2010), suggesting that it is unlikely that antibodies to *Neu5Gc* alone explain the observed immune activation. Moreover, whether cooking diminishes anti-*Neu5Gc* activity has not been studied. The mechanism of immune upregulation on the raw meat diet remains ripe for future inquiry, but our results do suggest that the adoption of cooking by ancestral hominins likely facilitated the consumption of a high-meat diet, another innovation argued to have been transformative in human evolution (Aiello and Wheeler 1995; Stanford and Bunn 2001).

In the case of tuber, we found that genes involved in carbohydrate metabolic processes were less highly expressed on cooked compared with raw tuber diets. This is consistent with an established literature showing that cooking enhances the efficiency of carbohydrate digestion by gelatinizing starch, a process that renders starch more susceptible to digestion by salivary and pancreatic amylases (Carmody and Wrangham 2009). Increases in the copy number of salivary amylase (*AMY1*) in modern human populations and pancreatic amylase (*AMY2B*) in domestic dogs—both of which have relatively starch-rich diets—have been hypothesized to reflect selection for increased expression to improve the digestion of starch-rich foods (Perry et al. 2007; Axelsson et al. 2013). In agreement with this we found that expression levels of *Amy1* were higher where diets imposed a higher demand for starch digestion, including in mice fed raw versus cooked tuber, and in mice fed tuber versus meat.

Genes that were differentially expressed with food type and food preparation in mice overlapped to an extent beyond chance with genes known to differ in their expression between humans and nonhuman primates. Moreover, when matching factors under the assumption that humans consume more meat and cooked items than nonhuman primates, we observed a strong correspondence in the directionality of expression patterns between the mouse data set and the human and nonhuman primate data sets. This correspondence confirms that controlled feeding experiments in mice can usefully inform aspects of human and nonhuman primate dietary divergence (Somel et al. 2008; Carmody et al. 2011). Importantly, it also suggests that published differences between humans and nonhuman primates in liver gene expression may be partly confounded by diet.

Although food type and food preparation were each associated with significant changes in gene expression, we found that only cooking-related genes were enriched among genes with evidence of positive selection in the human lineage. Notably, six of seven of these putatively selected cooking-related genes represent immune genes observed to be downregulated in their expression on cooked versus raw meat diets. To date, most reports on the evolutionary effects of cooking have focused on the enhancement of energy gain through increased nutrient digestibility and reduced costs of digestion. However our new results indicate that habitual cooking would also have led to reduced energy spent on immune upregulation, especially if ancestral hominins were already exploiting meat routinely prior to the adoption of cooking, as the current archaeological record suggests (Ferraro et al. 2013; Zink and Lieberman 2016).

The timing of the adoption of cooking remains unclear, with biological indicators suggesting an early date around 2 Ma (Wrangham et al. 1999), archaeological evidence suggesting controlled fire at 1 Ma (Berna et al. 2012) and hearths at 300,000 years ago (Shahack-Gross et al. 2014) although not all Neandertal occupations bear evidence of fire until approximately 40,000 years ago (Sandgathe et al. 2011), and the earliest direct evidence of cooked food consumption at just 50,000 years ago (Henry et al. 2011). In our data set, putatively selected cooking-related genes all predate the split between the human and Neandertal–Denisovan lineages, an event dated to between 275,000 and 765,000 years ago (Prufer et al. 2014). These new data support the view that 1) cooking predated the evolution of modern humans; and 2) cooking was practiced sufficiently often to have had selective effects in Neandertals and Denisovans, despite the sporadic archaeological evidence of fire (Roebroeks and Villa 2011).

Overall, our results draw new attention to the potentially transformative role of cooking for energy balance and food choice during human evolution. In addition, they support the idea that cooking was present among multiple hominin taxa at a date earlier than the earliest direct evidence of cooking in the archaeological record. Future work exploring the effects of a cooked diet at the molecular level will illuminate the human dietary niche and could ultimately provide a mechanistic understanding of the diverse positive and negative consequences of cooking for human health.

## Supplementary Material

Supplementary tables references, S1–S5, data S1, figures S1–S3 are available at *Genome Biology and Evolution* online (http://www.gbe.oxfordjournals.org/).

Supplementary Data
