# Supplementary material for: Genetic Evidence of Human Adaptation to a Cooked Diet
Source: Genome Biol Evol. 2016 Mar 15;8(4):1091–103. doi: 10.1093/gbe/evw059 (PMC4860691; doi:10.1093/gbe/evw059)
Supplement: Supplementary Data [file supp_8_4_1091__index.html]

Genetic Evidence of Human Adaptation to a Cooked Diet — Supplementary Data 

# Genetic Evidence of Human Adaptation to a Cooked Diet

## Supplementary Data

files

- Supplementary Data - docx file
- Supplementary Data - pdf file
