# Supplementary material for: Genetic Evidence of Human Adaptation to a Cooked Diet
Source: Genome Biol Evol. 2016 Mar 15;8(4):1091–103. doi: 10.1093/gbe/evw059 (PMC4860691; doi:10.1093/gbe/evw059)
Supplement: Supplementary Data [file supp_evw059_SI_021416_FINAL.docx]

**SUPPLEMENTARY FIGURES**


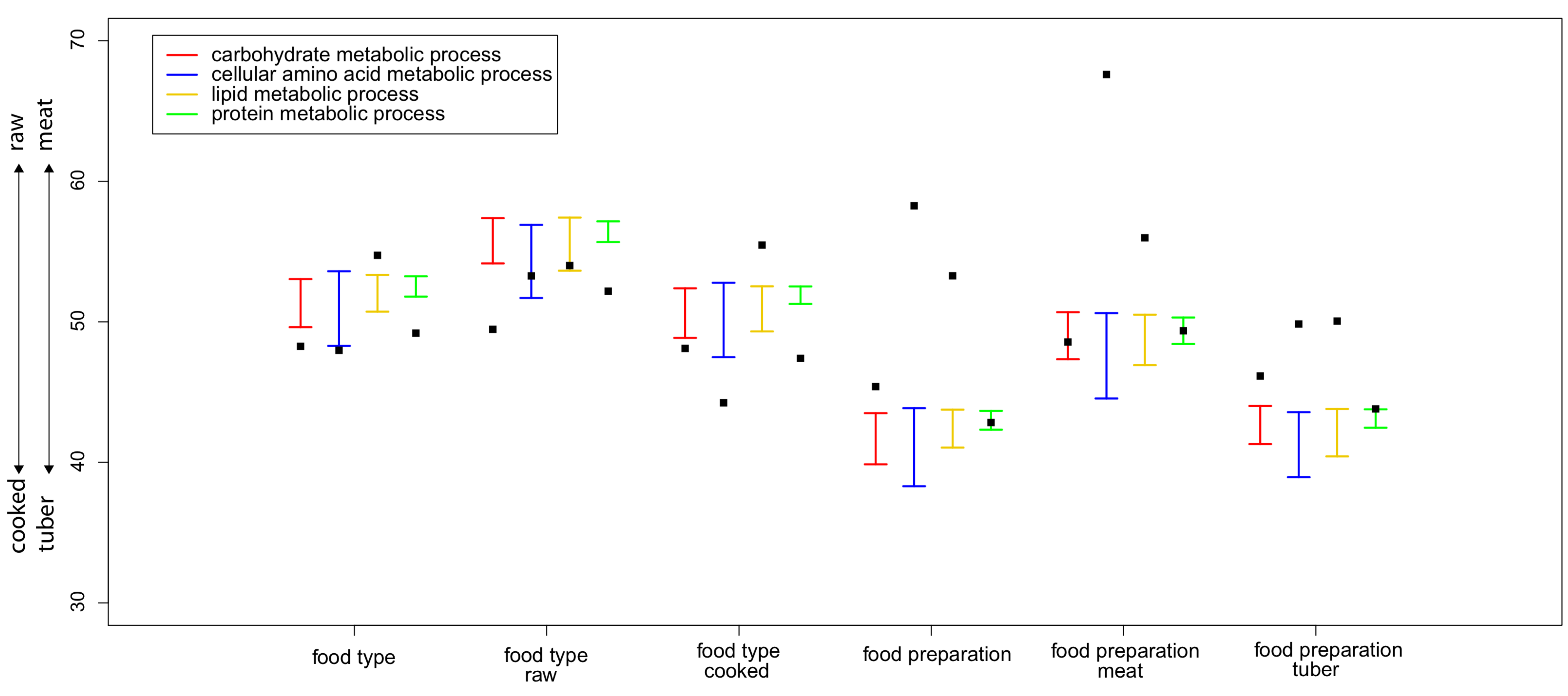


**Supplementary Figure 1. Macronutrient-specific gene expression patterns.** We assigned the sets of genes showing differential expression for each of the experimental factors (x-axis) to one of four metabolic processes defined by the Gene Ontology ([Gene Ontology Consortium 2000](#_ENREF_5)) [carbohydrate metabolic process (red), cellular amino acid metabolic process (blue), lipid metabolic process (yellow), and protein metabolic process (green)]. We then computed for each combination of gene set and category the percentage (y-axis) of genes showing increased expression on the meat (for food type) and raw (for food preparation) diets and plotted these as black squares with their corresponding 95% CI in the color of the assigned category.

a

**
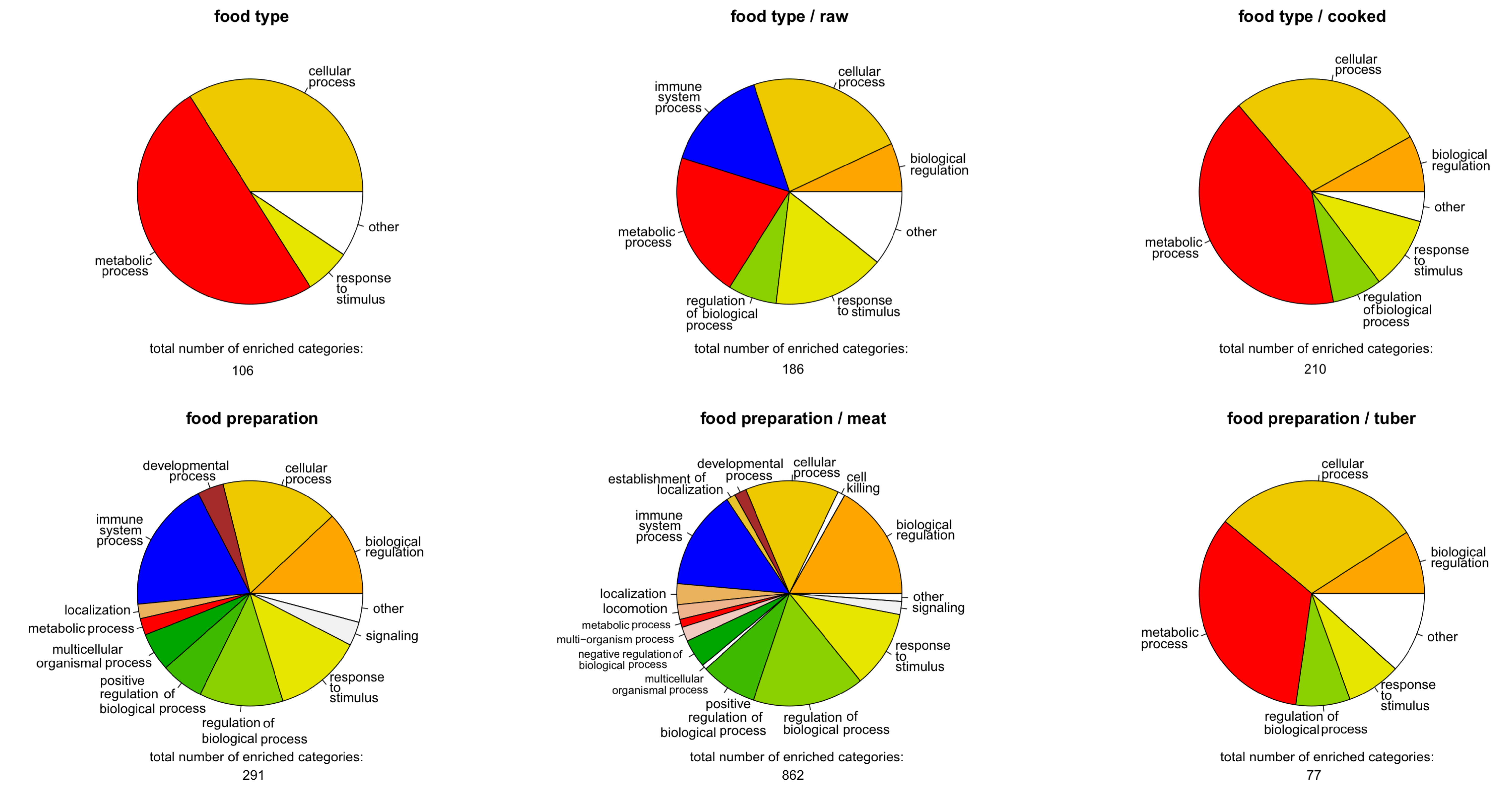
**

b


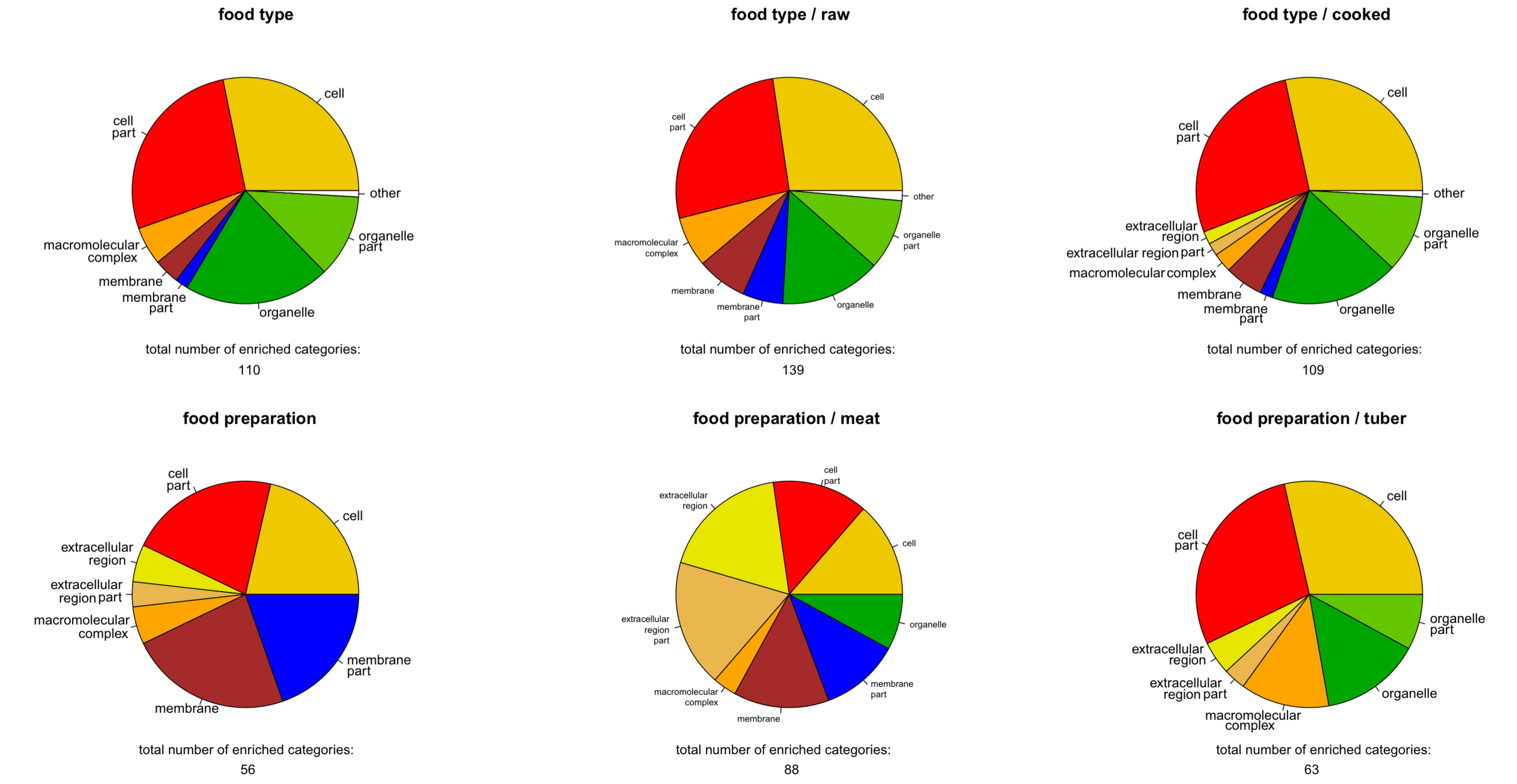


c

**
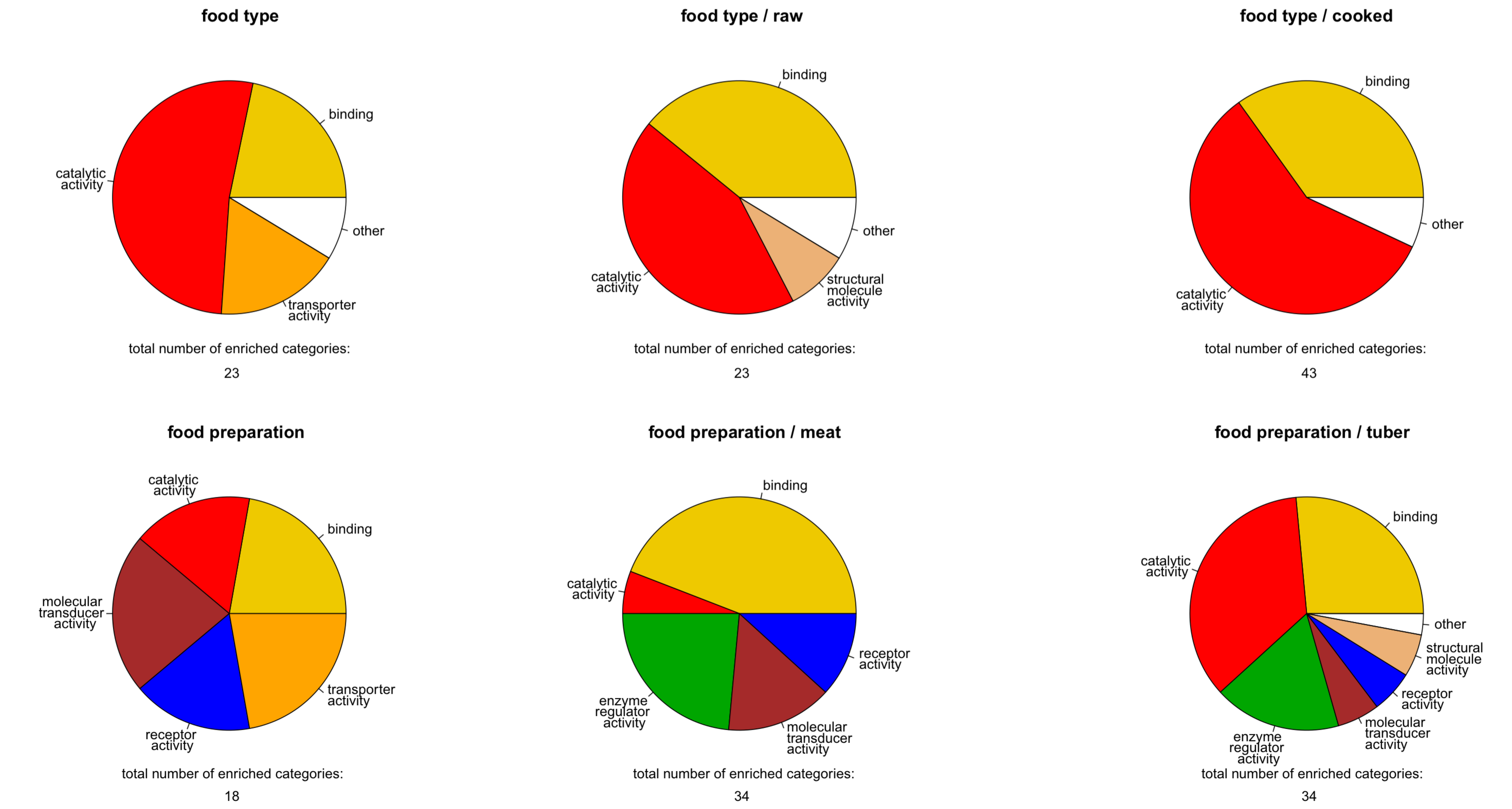
**

d

**
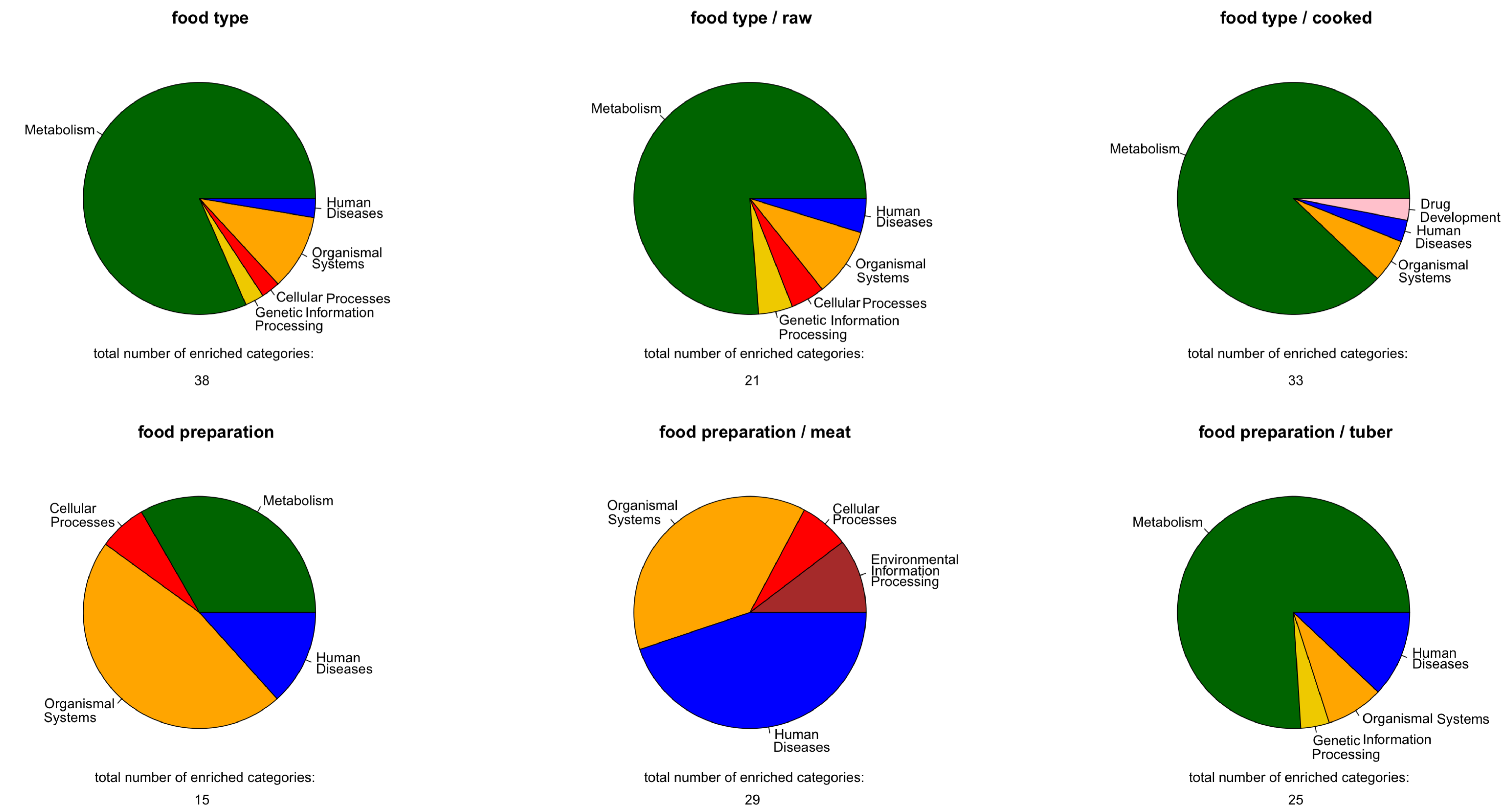
**

e

**
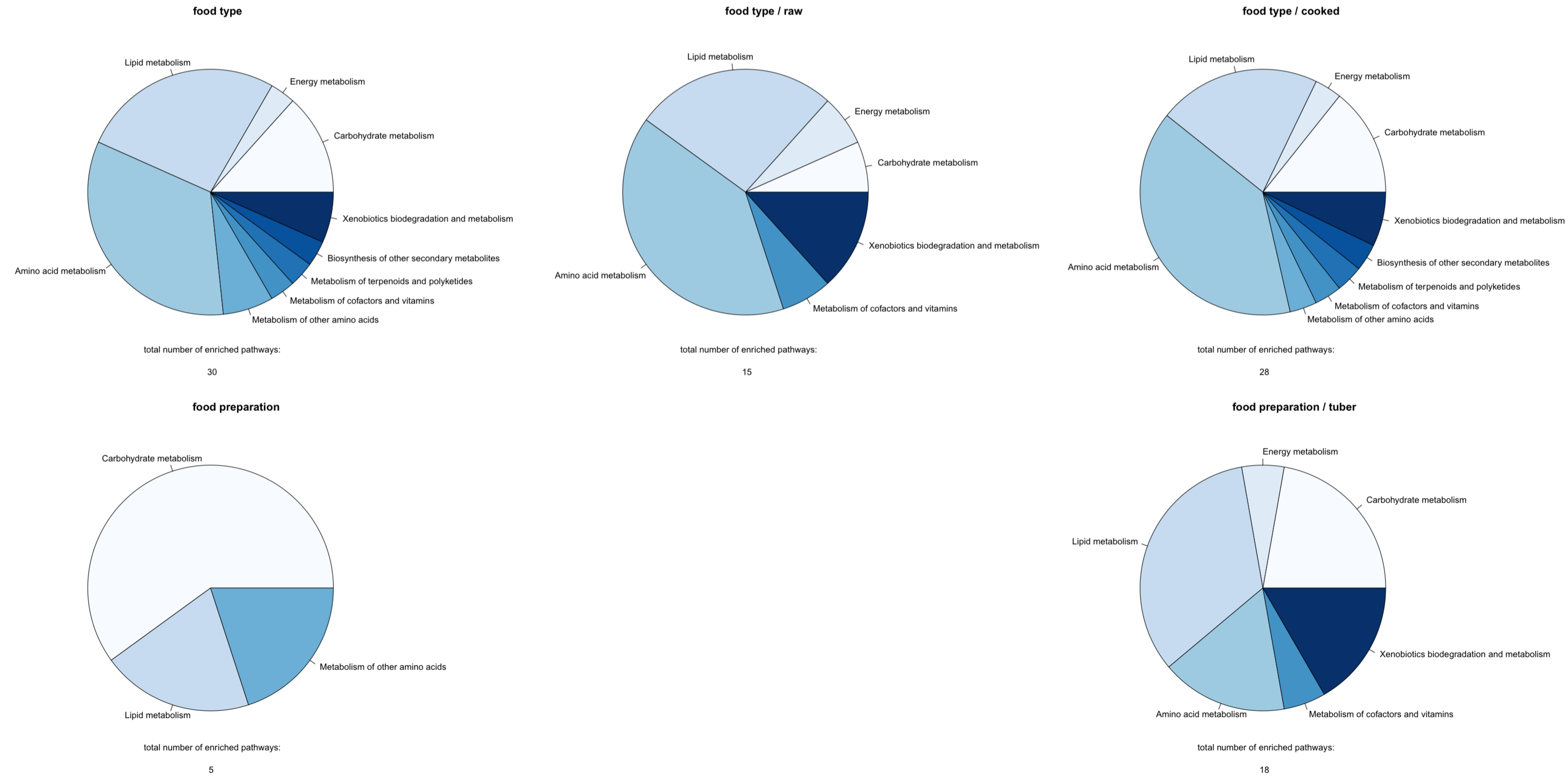
**

**Supplementary Figure 2. Functional enrichment.** Distribution of categories enriched in differentially expressed genes (p<0.05) in the Gene Ontology ([Gene Ontology Consortium 2000](#_ENREF_5)) for (**a**) biological processes, (**b**) cellular components, and (**c**) molecular functions, and in the KEGG pathway database ([Kanehisa and Goto 2000](#_ENREF_7)) for (**d**) all KEGG pathways and (**e**) all metabolic pathways. Each significant category is assigned to the second-highest term, i.e. categories directly under the root node of the respective ontology.

a


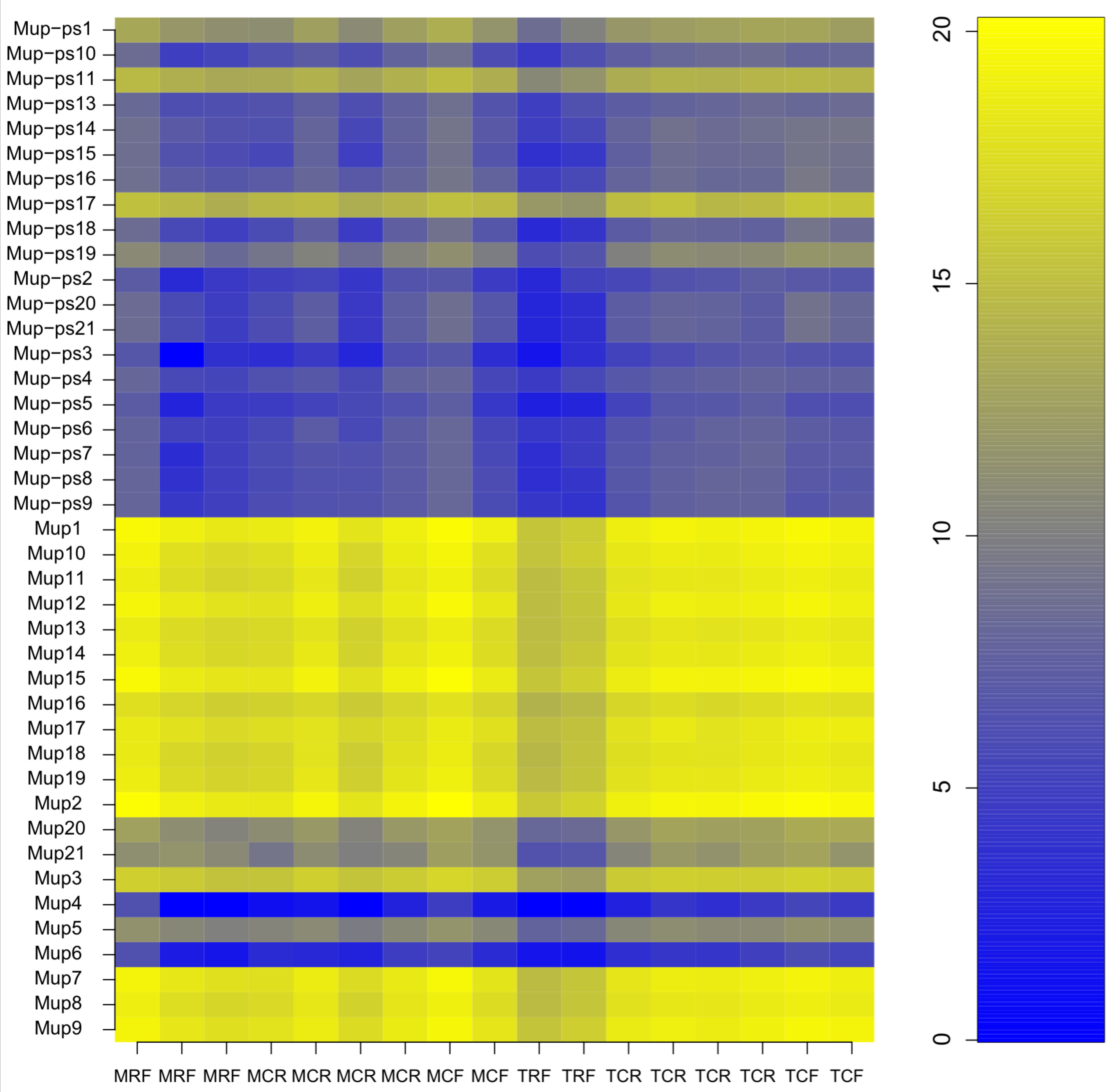


b


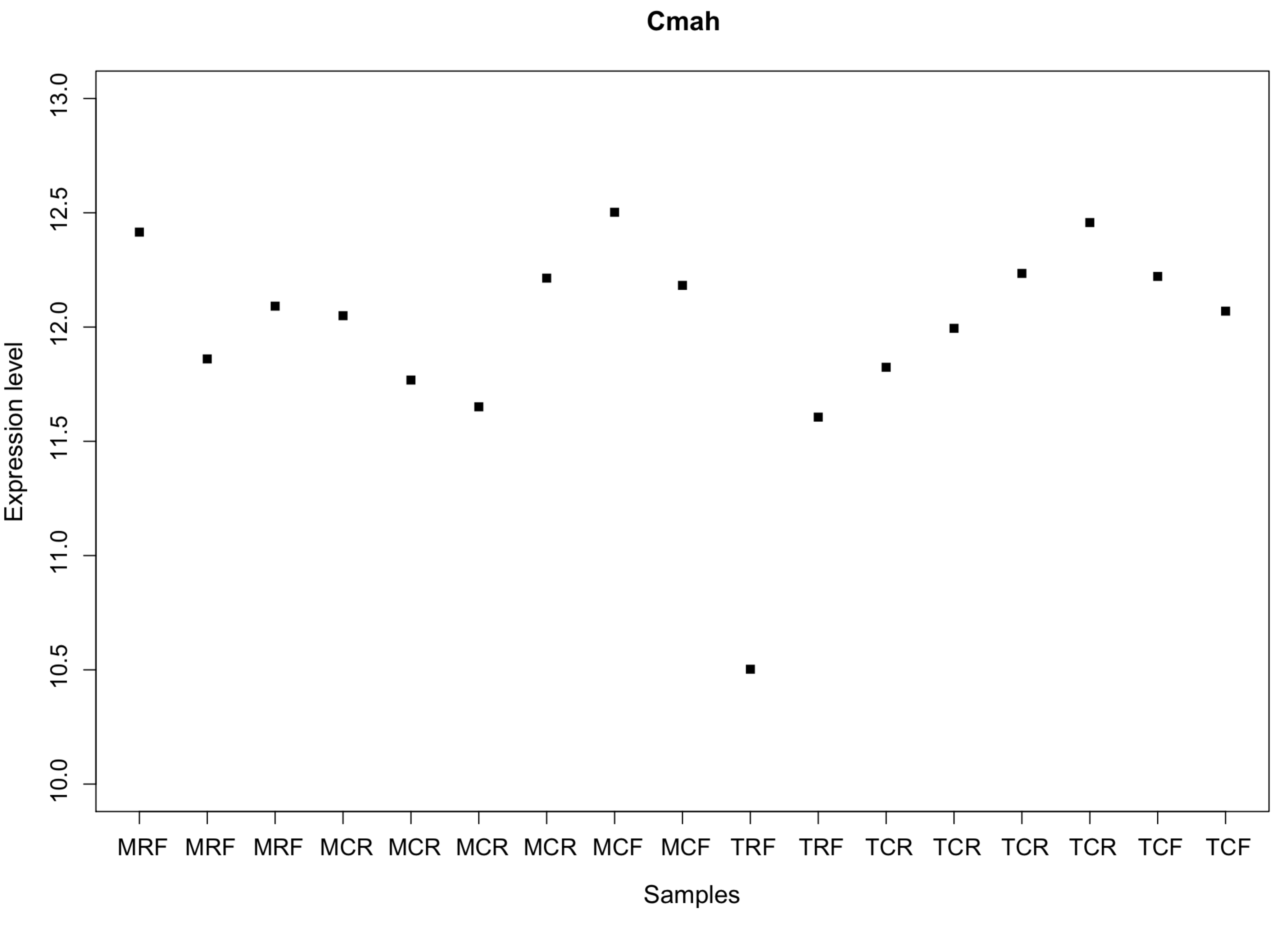


**Supplementary Figure 3.** **Cooking-related genes with evidence of pseudogenization in the human lineage.** (**a**) Intensity plot for the expression in liver of genes belonging to the Major urinary protein (*Mup*) gene family, and (**b**) expression of CMP-sialic acid hydroxylase (*Cmah*) in mice fed raw meat (MRF), cooked meat (MCF or MCR), raw tuber (TRF) or cooked tuber (TCF or TCR) diets. Expression counts were normalized ([Anders and Huber 2010](#_ENREF_1)) and log-transformed read counts were z-transformed. Colors in (**a**) correspond to the z-transformed expression values.

**SUPPLEMENTARY TABLES**

**Supplementary Table 1.** Nutritional composition of meat and tuber diets, per 100 g. MRF = meat/raw/free; MCF = meat/cooked/free; MCR = meat/cooked/restricted; TRF = tuber/raw/free; TCF = tuber/cooked/free; TCR = tuber/cooked/restricted. All n=3; mean±SEM.

| Fresh-weight | **Meat** | | | **Tuber** | | |
| --- | --- | --- | --- | --- | --- | --- |
|  | MRF | MCF | MCR | TRF | TCF | TCR |
| Water (g) ^a^ | 73.2 ± 0.7 | 64.8 ± 0.7 | 69.1 ± 1.1 | 80.9 ± 0.6 | 70.4 ± 0.2 | 70.5 ± 0.1 |
| Protein (g) ^b^ | 22.4 ± 0.3 | 29.9 ± 0.3 | 27.1 ± 0.4 | 0.9 ± 0.1 | 1.4 ± 0.1 | 1.2 ± 0.1 |
| Lipid (g) ^c^ | 3.4 ± 0.4 | 3.6 ± 0.1 | 3.5 ± 0.1 | 0.3 ± 0.1 | 0.2 ± 0.0 | 0.1 ± 0.0 |
| Fiber (g) ^d^ | − | − | − | 1.7 ± 0.1 | 1.5 ± 0.1 | 1.6 ± 0.2 |
| Ash (g) ^e^ | 1.1 ± 0.1 | 1.5 ± 0.1 | 1.4 ± 0.0 | 0.7 ± 0.1 | 1.0 ± 0.0 | 0.9 ± 0.0 |
| Carbohydrate (g) ^f^ | -0.2 ± 1.3 | 0.2 ± 0.8 | -1.1 ± 1.1 | 15.5 ± 0.8 | 25.5 ± 0.1 | 25.7 ± 0.2 |
| Total starch (g) ^g^ | − | − | − | 8.4 ± 0.5 | 6.4 ± 0.3 | 6.7 ± 0.6 |
| ME (kcal) ^h^ | 120 ± 1 | 153 ± 3 | 135 ± 5 | 68 ± 3 | 109 ± 1 | 109 ± 1 |
| Dry-weight | Meat | | | Tuber | | |
|  | MRF | MCF | MCR | TRF | TCF | TCR |
| Water | 0 | 0 | 0 | 0 | 0 | 0 |
| Protein | 83.7 ± 1.0 | 84.9 ± 0.8 | 87.7 ± 1.3 | 4.6 ± 0.5 | 4.7 ± 0.3 | 4.1 ± 0.3 |
| Lipid | 12.8 ± 1.7 | 10.1 ± 0.3 | 11.5 ± 0.4 | 1.4 ± 0.4 | 0.6 ± 0.1 | 0.4 ± 0.1 |
| Fiber | − | − | − | 9.0 ± 0.6 | 5.2 ± 0.3 | 5.4 ± 0.1 |
| Ash | 4.1 ± 0.3 | 4.2 ± 0.4 | 4.6 ± 0.1 | 3.6 ± 0.3 | 3.4 ± 0.2 | 3.1 ± 0.1 |
| Carbohydrate | -0.6 ± 2.0 | 0.8 ± 0.9 | -3.8 ± 1.4 | 81.4 ± 0.9 | 86.1 ± 0.5 | 87.0 ± 0.3 |
| Total starch | − | − | − | 44.0 ± 2.0 | 21.6 ± 1.3 | 22.6 ± 1.2 |
| ME (kcal) | 448 ± 3 | 444 ± 1 | 439 ± 2 | 357 ± 2 | 369 ± 1 | 368 ± 1 |

Notes:

− Indicates value below detection threshold of the biochemical assay.

^a^ Water content determined as grams of sample lost after freeze-drying to constant mass and hot weighing the residue at 100˚C.

^b^ Crude protein content determined by Kjeldahl procedure for total nitrogen ([Pierce and Haenisch 1947](#_ENREF_9)).

^c^ Total lipid content determined by petroleum ether extraction ([Conklin-Brittain, et al. 2006](#_ENREF_4)).

^d^ Neutral detergent fiber content determined using Detergent System of Fiber Analysis ([Robertson and van Soest 1980](#_ENREF_11)).

^e^ Ash determined by incinerating sample in a muffle furnace at 500˚C and hot weighing residue at 100˚C.

^f^ Total non-structural carbohydrate determined by difference: Carbohydrate (g) = 100 g – Water (g) – Protein (g) – Lipid (g) – Fiber (g) – Ash (g). Since this value is determined by subtraction, negative values are possible for samples with negligible carbohydrate content.

^g^ Total starch determined using an amyloglucosidase/α-amylase kit (Total Starch Kit, Megazyme).

^h^ Metabolizable energy (ME) calculated from macronutrient content using conventional factors: 4 kcal/g for crude protein, 9 kcal/g for total lipid, and 4 kcal/g for total non-structural carbohydrate.

**Supplementary Table 2.** Expression skew among differentially expressed genes.

| **Factor** | **Number of genes with increased expression (FDR<0.05)** |
| --- | --- |
| Food type | Meat: 1,543  Tuber: 1,403 |
| Food type – raw | Meat: 275  Tuber: 203 |
| Food type – cooked | Meat: 803  Tuber: 599 |
| Food preparation | Raw: 71  Cooked: 52 |
| Food preparation – meat | Raw: 110  Cooked: 2 |
| Food preparation – tuber | Raw: 161  Cooked: 283 |

**Supplementary Table 3.** Odds ratios and corresponding p-values and false discovery rates (FDR) for genes with analogous fold changes in the present study versus two related studies.

| **Factors** | **Overlap with dataset reporting microarray-based liver expression in mice fed human versus chimpanzee diets (**[**Somel, et al. 2008**](#_ENREF_12)**)** | **Overlap with dataset reporting RNAseq-based liver expression in human versus non-human primates (**[**Blekhman, et al. 2010**](#_ENREF_2)**)** |
| --- | --- | --- |
| Food type | 1.8 (p=4.7e-25, FDR=7.1e-25) | 2.0 (p=1.4e-25, FDR=8.4e-25) |
| Food type – raw | 8.5 (p=5.6e-37, FDR=1,1e-36) | 1.0 (p=0.40, FDR=0.48) |
| Food type – cooked | 4.1 (p=6.1e-67, FDR=3.7e-66) | 1.4 (p=8.8e-6, FDR=2.6e-5) |
| Food preparation | 35.0 (p=9.1e-21, FDR=1.1e-20) | 2.6 (p=4.9e-3, FDR=9.8e-3) |
| Food preparation – meat | 5.8 (p=0.02, FDR=0.02) | 0.4 (p=0.98, FDR=0.98) |
| Food preparation – tuber | 13.3 (p=7.1e-59, FDR=2.1e-58) | 1.3 (p=0.10, FDR=0.15) |

**Supplementary Table 4.** **(a)** Overlap of differentially expressed genes and positively selected genes on the human or chimpanzee lineages since our last common ancestor ([Kosiol, et al. 2008](#_ENREF_8)). **(b)** Number of differentially expressed genes with evidence of positive selection in their promoter regions on the human or chimpanzee lineages since our last common ancestor ([Haygood, et al. 2007](#_ENREF_6)). Data reflect the number of genes or promoters, respectively, with odds ratio (OR), p-value (p) and false discovery rate (FDR). Odds ratios exceeding those expected by chance are indicated by asterisks (*** p<0.001; * p<0.05).

a

| **Factors** | **Human lineage  (total positively selected genes: 152)** | **Chimpanzee lineage  (total positively selected genes: 118)** |
| --- | --- | --- |
| Food type | 20 (OR=0.8, p=0.86, FDR=0.86) | 12 (OR=0.6, p=0.97, FDR=0.97) |
| Food type – raw | 3 (OR=0.75, p=0.76, FDR=0.86) | 5 (OR=1.7, p=0.20, FDR=0.30) |
| Food type – cooked | 10 (OR=0.9, p=0.73, FDR=0.86) | 7 (OR=0.8, p=0.80, FDR=0.96) |
| Food preparation | 4 (OR=4.0, p=0.02, FDR=0.06)* | 2 (OR=2.6, p=0.18, FDR=0.30) |
| Food preparation – meat | 6 (OR=6.7, p=4.3e-4, FDR=2.6e-3)*** | 2 (OR=2.8, p=0.16, FDR=0.30) |
| Food preparation – tuber | 3 (OR=0.8, p=0.71, FDR=0.86) | 6 (OR=2.2, p=0.07, FDR=0.30) |

b

| **Factors** | **Human lineage (total: 390)** | **Chimpanzee lineage (total: 412)** |
| --- | --- | --- |
| Food type | 57 (OR=0.9, p=0.79, FDR=0.79) | 56 (OR=0.8, p=0.92, FDR=1) |
| Food type – raw | 14 (OR=1.4, p=0.15, FDR=0.66) | 9 (OR=0.8, p=0.74, FDR=1) |
| Food type – cooked | 32 (OR=1.1, p=0.36, FDR=0.72) | 26 (OR=0.8, p=0.86, FDR=1) |
| Food preparation | 2 (OR=0.8, p=0.74, FDR=0.79) | 0 (OR=0, p=1.00, FDR=1) |
| Food preparation – meat | 4 (OR=1.7, p=0.22, FDR=0.66) | 3 (OR=1.2, p=0.46, FDR=1) |
| Food preparation – tuber | 7 (OR=0.7, p=0.74, FDR=0.79) | 4 (OR=0.4, p=0.99, FDR=1) |

**Supplementary Table 5.** Number of differentially expressed genes with evidence of positive selection on the human lineage since the split from Neandertals and Denisovans ([Prufer, et al. 2014](#_ENREF_10)). Data reflect the number of genes with odds ratio (OR), p-value (p) and false discovery rate (FDR).

| **Factors in our study** | **Human lineage (total: 195)** |
| --- | --- |
| Food type | 15 (OR=0.4, p=1.00, FDR=1) |
| Food type – raw | 2 (OR=0.4, p=0.96, FDR=1) |
| Food type – cooked | 10 (OR=0.7, p=0.93, FDR=1) |
| Food preparation | 0 (OR=0, p=1.00, FDR=1) |
| Food preparation – meat | 0 (OR=0, p=1.00, FDR=1) |
| Food preparation – tuber | 2 (OR=0.4, p=0.95, FDR=1) |

**SUPPLEMENTARY DATA**

Uploaded as separate file.

**SUPPLEMENTARY REFERENCES**

Anders S, Huber W 2010. Differential expression analysis for sequence count data. Genome Biology 11: R106.

Blekhman R, Marioni JC, Zumbo P, Stephens M, Gilad Y 2010. Sex-specific and lineage-specific alternative splicing in primates. Genome Research 20: 180-189.

Cole JR, et al. 2005. The Ribosomal Database Project (RDP-II): sequences and tools for high-throughput rRNA analysis. Nucleic Acids Research 33: D294-D296.

Conklin-Brittain NL, Knott CD, Wrangham RW. 2006. Energy intake by wild chimpanzees and orangutans: methodological considerations and a preliminary comparison. In: Hohmann G, Robbins MM, Boesch C, editors. Feeding Ecology in Apes and Other Primates: Ecological, Physical and Behavioral Aspects. Cambridge: Cambridge University Press. p. 445-471.

Gene Ontology Consortium 2000. Gene Ontology: tool for the unification of biology. Nature Genetics 25: 25-29.

Haygood R, Fedrigo O, Hanson B, Yokoyama KD, Wray GA 2007. Promoter regions of many neural- and nutrition-related genes have experienced positive selection during human evolution. Nature Genetics 39: 1140–1144.

Kanehisa M, Goto S 2000. KEGG: Kyoto Encyclopedia of Genes and Genomes. Nucleic Acids Research 28: 27-30.

Kosiol C, et al. 2008. Patterns of positive selection in six mammalian genomes. PLoS Genetics 4: e1000144.

Pierce WC, Haenisch EL. 1947. Quantitative Analysis. New York: John Wiley & Sons.

Prufer K, et al. 2014. The complete genome sequence of a Neanderthal from the Altai Mountains. Nature 505: 43-49.

Robertson JB, van Soest PJ. 1980. The detergent system of analysis and its application to human foods. In: James WPT, Theander O, editors. The Analysis of Dietary Fiber in Food. New York: Marcel Dekker. p. 123-158.

Somel M, et al. 2008. Human and chimpanzee gene expression differences replicated in mice fed different diets. PLoS One 3: e1504.
