# Supplementary material for: Genetic Evidence of Human Adaptation to a Cooked Diet
Source: Genome Biol Evol. 2016 Mar 15;8(4):1091–103. doi: 10.1093/gbe/evw059 (PMC4860691; doi:10.1093/gbe/evw059)
Supplement: Supplementary Data [file supp_evw059_SuppDataS1.pdf]

GO domain	GO term	FWER
Meat vs. Tuber (Cooked and Raw combined)		
molecular function	structural constituent of ribosome	<0.001
molecular function	catalytic activity	<0.001
molecular function	monooxygenase activity	<0.001
molecular function	iron ion binding	<0.001
molecular function	transaminase activity	<0.001
molecular function	electron carrier activity	<0.001
molecular function	oxidoreductase activity	<0.001
molecular function	oxidoreductase activity, acting on CH-OH group of donors	<0.001
molecular function	oxidoreductase activity, acting on the CH-OH group of donors, NAD or NADP as acceptor	<0.001
molecular function	oxidoreductase activity, acting on paired donors, with incorporation or reduction of molecular oxygen	<0.001
molecular function	transferase activity, transferring alkyl or aryl (other than methyl) groups	<0.001
molecular function	transferase activity, transferring nitrogenous groups	<0.001
molecular function	lyase activity	<0.001
molecular function	vitamin binding	<0.001
molecular function	heme binding	<0.001
molecular function	carboxylic acid binding	<0.001
molecular function	tetrapyrrole binding	<0.001
molecular function	cofactor binding	<0.001
molecular function	coenzyme binding	<0.001
biological process	monosaccharide metabolic process	<0.001
biological process	glucose metabolic process	<0.001
biological process	alcohol metabolic process	<0.001
biological process	organic acid metabolic process	<0.001
biological process	cellular amino acid metabolic process	<0.001
biological process	lipid metabolic process	<0.001
biological process	fatty acid metabolic process	<0.001
biological process	fatty acid beta-oxidation	<0.001
biological process	steroid biosynthetic process	<0.001
biological process	coenzyme metabolic process	<0.001

biological process	response to stress	<0.001
biological process	metabolic process	<0.001
biological process	steroid metabolic process	<0.001
biological process	cholesterol metabolic process	<0.001
biological process	lipid biosynthetic process	<0.001
biological process	catabolic process	<0.001
biological process	biosynthetic process	<0.001
biological process	fatty acid catabolic process	<0.001
biological process	cellular amino acid catabolic process	<0.001
biological process	glutamine family amino acid metabolic process	<0.001
biological process	response to endogenous stimulus	<0.001
biological process	response to hormone stimulus	<0.001
biological process	cellular process	<0.001
biological process	response to organic substance	<0.001
biological process	regulation of cellular ketone metabolic process	<0.001
biological process	lipid catabolic process	<0.001
biological process	organic acid biosynthetic process	<0.001
biological process	organic acid catabolic process	<0.001
biological process	sterol metabolic process	<0.001
biological process	sterol biosynthetic process	<0.001
biological process	regulation of fatty acid metabolic process	<0.001
biological process	hexose metabolic process	<0.001
biological process	fatty acid oxidation	<0.001
biological process	carboxylic acid metabolic process	<0.001
biological process	lipid modification	<0.001
biological process	monocarboxylic acid metabolic process	<0.001
biological process	lipid oxidation	<0.001
biological process	cellular ketone metabolic process	<0.001
biological process	response to chemical stimulus	<0.001
biological process	oxoacid metabolic process	<0.001
biological process	cellular metabolic process	<0.001
biological process	primary metabolic process	<0.001
biological process	cellular lipid catabolic process	<0.001
biological process	cellular catabolic process	<0.001
biological process	cellular lipid metabolic process	<0.001
biological process	small molecule metabolic process	<0.001
biological process	small molecule catabolic process	<0.001
biological process	small molecule biosynthetic process	<0.001
biological process	regulation of fatty acid oxidation	<0.001
biological process	carboxylic acid biosynthetic process	<0.001

biological process	carboxylic acid catabolic process	<0.001
biological process	glycerolipid metabolic process	<0.001
biological process	cofactor metabolic process	<0.001
biological process	response to glucocorticoid stimulus	<0.001
biological process	oxidation-reduction process	<0.001
biological process	organic substance transport	<0.001
biological process	monocarboxylic acid catabolic process	<0.001
cellular component	cell fraction	<0.001
cellular component	intracellular	<0.001
cellular component	cell	<0.001
cellular component	soluble fraction	<0.001
cellular component	cytoplasm	<0.001
cellular component	mitochondrion	<0.001
cellular component	peroxisome	<0.001
cellular component	endoplasmic reticulum	<0.001
cellular component	cytosol	<0.001
cellular component	cytosolic ribosome	<0.001
cellular component	microbody	<0.001
cellular component	organelle	<0.001
cellular component	membrane-bounded organelle	<0.001
cellular component	intracellular organelle	<0.001
cellular component	intracellular membrane-bounded organelle	<0.001
cellular component	intracellular part	<0.001
cellular component	endoplasmic reticulum part	<0.001
cellular component	cytoplasmic part	<0.001
cellular component	cell part	<0.001
biological process	aromatic compound catabolic process	0.001
cellular component	mitochondrial envelope	0.001
cellular component	cytosolic large ribosomal subunit	0.001
cellular component	vesicular fraction	0.001
cellular component	ribosomal subunit	0.001
cellular component	mitochondrial part	0.001
biological process	acylglycerol metabolic process	0.002
biological process	isoprenoid biosynthetic process	0.002
biological process	response to metal ion	0.002
biological process	response to corticosteroid stimulus	0.002
cellular component	microsome	0.002
cellular component	mitochondrial membrane	0.002
molecular function	palmitoyl-CoA hydrolase activity	0.003

molecular function	oxidoreductase activity, acting on paired donors, with incorporation or reduction of molecular oxygen, NADH or NADPH as one donor, and incorporation of one atom of oxygen	0.003
molecular function	pyridoxal phosphate binding	0.003
molecular function	vitamin B6 binding	0.003
cellular component	endoplasmic reticulum lumen	0.003
molecular function	serine-type endopeptidase inhibitor activity	0.004
biological process	neutral lipid metabolic process	0.004
cellular component	ribosome	0.006
cellular component	organelle membrane	0.006
cellular component	intracellular organelle part	0.006
cellular component	mitochondrial matrix	0.008
molecular function	acetyl-CoA C-acyltransferase activity	0.009
biological process	response to xenobiotic stimulus	0.009
biological process	phosphatidylcholine metabolic process	0.009
cellular component	endoplasmic reticulum membrane	0.009
biological process	very long-chain fatty acid metabolic process	0.011
biological process	carbohydrate metabolic process	0.011
biological process	triglyceride metabolic process	0.011
biological process	cholesterol biosynthetic process	0.011
biological process	isoprenoid metabolic process	0.011
biological process	cellular amino acid biosynthetic process	0.011
biological process	glutamine family amino acid biosynthetic process	0.011
biological process	response to extracellular stimulus	0.011
biological process	organophosphate metabolic process	0.011
biological process	response to nutrient levels	0.011
biological process	ethanolamine-containing compound metabolic process	0.011
biological process	response to peptide hormone stimulus	0.011
biological process	glycerolipid catabolic process	0.011
cellular component	cytosolic small ribosomal subunit	0.012
biological process	cellular modified amino acid metabolic process	0.013
biological process	cellular aromatic compound metabolic process	0.015
cellular component	nuclear outer membrane-endoplasmic reticulum membrane network	0.015
molecular function	glutathione transferase activity	0.017
molecular function	oxidoreductase activity, acting on the CH-NH group of donors	0.017
biological process	response to inorganic substance	0.017

biological process	energy derivation by oxidation of organic compounds	0.017
biological process	cellular amine metabolic process	0.017
biological process	regulation of glycogen metabolic process	0.019
biological process	cellular biogenic amine metabolic process	0.02
biological process	regulation of polysaccharide metabolic process	0.021
biological process	glycogen biosynthetic process	0.022
biological process	generation of precursor metabolites and energy	0.022
biological process	glucan biosynthetic process	0.022
molecular function	fatty-acyl-CoA binding	0.023
cellular component	extracellular space	0.029
cellular component	organelle part	0.029
biological process	response to topologically incorrect protein	0.03
biological process	cellular biosynthetic process	0.032
biological process	xenobiotic metabolic process	0.035
biological process	positive regulation of fatty acid metabolic process	0.035
biological process	cellular response to xenobiotic stimulus	0.035
molecular function	carbon-carbon lyase activity	0.041
biological process	porphyrin-containing compound metabolic process	0.043
molecular function	enzyme inhibitor activity	0.049
Meat vs. Tuber (Cooked)		
molecular function	catalytic activity	<0.001
molecular function	glutathione transferase activity	<0.001
molecular function	monooxygenase activity	<0.001
molecular function	iron ion binding	<0.001
molecular function	electron carrier activity	<0.001
molecular function	oxidoreductase activity	<0.001
molecular function	oxidoreductase activity, acting on CH-OH group of donors	<0.001
molecular function	oxidoreductase activity, acting on the CH-OH group of donors, NAD or NADP as acceptor	<0.001
molecular function	oxidoreductase activity, acting on paired donors, with incorporation or reduction of molecular oxygen	<0.001
molecular function	lyase activity	<0.001
molecular function	vitamin binding	<0.001
molecular function	heme binding	<0.001
molecular function	tetrapyrrole binding	<0.001

molecular function	cofactor binding	<0.001
molecular function	coenzyme binding	<0.001
biological process	carbohydrate metabolic process	<0.001
biological process	monosaccharide metabolic process	<0.001
biological process	glucose metabolic process	<0.001
biological process	alcohol metabolic process	<0.001
biological process	organic acid metabolic process	<0.001
biological process	generation of precursor metabolites and energy	<0.001
biological process	cellular amino acid metabolic process	<0.001
biological process	cellular modified amino acid metabolic process	<0.001
biological process	lipid metabolic process	<0.001
biological process	fatty acid metabolic process	<0.001
biological process	fatty acid beta-oxidation	<0.001
biological process	neutral lipid metabolic process	<0.001
biological process	acylglycerol metabolic process	<0.001
biological process	triglyceride metabolic process	<0.001
biological process	steroid biosynthetic process	<0.001
biological process	cholesterol biosynthetic process	<0.001
biological process	coenzyme metabolic process	<0.001
biological process	response to stress	<0.001
biological process	metabolic process	<0.001
biological process	steroid metabolic process	<0.001
biological process	cholesterol metabolic process	<0.001
biological process	isoprenoid biosynthetic process	<0.001
biological process	lipid biosynthetic process	<0.001
biological process	catabolic process	<0.001
biological process	fatty acid catabolic process	<0.001
biological process	cellular amino acid catabolic process	<0.001
biological process	glutamine family amino acid metabolic process	<0.001
biological process	response to endogenous stimulus	<0.001
biological process	response to hormone stimulus	<0.001
biological process	response to organic substance	<0.001
biological process	regulation of cellular ketone metabolic process	<0.001
biological process	regulation of glucose metabolic process	<0.001
biological process	energy derivation by oxidation of organic compounds	<0.001
biological process	lipid catabolic process	<0.001
biological process	carbohydrate biosynthetic process	<0.001
biological process	organic acid biosynthetic process	<0.001
biological process	organic acid catabolic process	<0.001

biological process	sterol metabolic process	<0.001
biological process	sterol biosynthetic process	<0.001
biological process	hexose metabolic process	<0.001
biological process	fatty acid oxidation	<0.001
biological process	aromatic compound catabolic process	<0.001
biological process	carboxylic acid metabolic process	<0.001
biological process	lipid modification	<0.001
biological process	monocarboxylic acid metabolic process	<0.001
biological process	lipid oxidation	<0.001
biological process	cellular ketone metabolic process	<0.001
biological process	response to chemical stimulus	<0.001
biological process	response to peptide hormone stimulus	<0.001
biological process	oxoacid metabolic process	<0.001
biological process	cellular metabolic process	<0.001
biological process	primary metabolic process	<0.001
biological process	cellular lipid catabolic process	<0.001
biological process	cellular catabolic process	<0.001
biological process	cellular lipid metabolic process	<0.001
biological process	cellular carbohydrate metabolic process	<0.001
biological process	small molecule metabolic process	<0.001
biological process	small molecule catabolic process	<0.001
biological process	small molecule biosynthetic process	<0.001
biological process	carboxylic acid biosynthetic process	<0.001
biological process	carboxylic acid catabolic process	<0.001
biological process	cofactor metabolic process	<0.001
biological process	oxidation-reduction process	<0.001
biological process	monocarboxylic acid catabolic process	<0.001
cellular component	cell fraction	<0.001
cellular component	extracellular region	<0.001
cellular component	soluble fraction	<0.001
cellular component	insoluble fraction	<0.001
cellular component	cytoplasm	<0.001
cellular component	mitochondrion	<0.001
cellular component	mitochondrial envelope	<0.001
cellular component	peroxisome	<0.001
cellular component	endoplasmic reticulum	<0.001
cellular component	endoplasmic reticulum lumen	<0.001
cellular component	endoplasmic reticulum membrane	<0.001
cellular component	microsome	<0.001
cellular component	organelle membrane	<0.001

cellular component	mitochondrial membrane	<0.001
cellular component	nuclear outer membrane-endoplasmic reticulum membrane network	<0.001
cellular component	microbody	<0.001
cellular component	vesicular fraction	<0.001
cellular component	membrane-bounded organelle	<0.001
cellular component	intracellular membrane-bounded organelle	<0.001
cellular component	intracellular part	<0.001
cellular component	mitochondrial part	<0.001
cellular component	endoplasmic reticulum part	<0.001
cellular component	cytoplasmic part	<0.001
molecular function	transaminase activity	0.001
molecular function	transferase activity, transferring alkyl or aryl (other than methyl) groups	0.001
molecular function	pyridoxal phosphate binding	0.001
molecular function	carboxylic acid binding	0.001
molecular function	vitamin B6 binding	0.001
biological process	energy reserve metabolic process	0.001
biological process	acyl-CoA metabolic process	0.001
biological process	isoprenoid metabolic process	0.001
biological process	aromatic amino acid family catabolic process	0.001
biological process	regulation of cellular carbohydrate metabolic process	0.001
biological process	regulation of lipid metabolic process	0.001
biological process	response to insulin stimulus	0.001
biological process	thioester metabolic process	0.001
biological process	regulation of carbohydrate biosynthetic process	0.001
biological process	dicarboxylic acid metabolic process	0.001
biological process	monosaccharide biosynthetic process	0.001
biological process	glycerolipid metabolic process	0.001
cellular component	mitochondrial inner membrane	0.001
molecular function	transferase activity, transferring nitrogenous groups	0.002
biological process	regulation of carbohydrate metabolic process	0.002
biological process	glutathione metabolic process	0.002
biological process	regulation of fatty acid metabolic process	0.002
biological process	response to lipid	0.002
cellular component	membrane fraction	0.002
cellular component	organelle inner membrane	0.002
biological process	peptide metabolic process	0.003

biological process	glutamine family amino acid biosynthetic process	0.003
biological process	amine metabolic process	0.003
biological process	carbohydrate catabolic process	0.003
biological process	hexose biosynthetic process	0.003
biological process	cellular respiration	0.003
biological process	positive regulation of lipid metabolic process	0.003
biological process	regulation of lipid biosynthetic process	0.003
biological process	cellular response to chemical stimulus	0.003
biological process	organic substance transport	0.003
cellular component	extracellular space	0.004
cellular component	intracellular	0.004
molecular function	oxidoreductase activity, acting on the CH-CH group of donors	0.005
molecular function	oxidoreductase activity, acting on paired donors, with incorporation or reduction of molecular oxygen, reduced flavin or flavoprotein as one donor, and incorporation of one atom of oxygen	0.005
biological process	gluconeogenesis	0.005
biological process	cellular response to lipid	0.005
molecular function	palmitoyl-CoA hydrolase activity	0.006
molecular function	L-amino acid transmembrane transporter activity	0.007
cellular component	melanosome	0.011
cellular component	pigment granule	0.011
biological process	glutamate metabolic process	0.012
biological process	response to oxidative stress	0.012
biological process	response to inorganic substance	0.012
biological process	response to metal ion	0.012
biological process	organophosphate metabolic process	0.012
biological process	negative regulation of cellular carbohydrate metabolic process	0.013
biological process	response to stilbenoid	0.013
biological process	cellular amine metabolic process	0.013
molecular function	carboxy-lyase activity	0.014
biological process	regulation of fatty acid oxidation	0.015
cellular component	organelle envelope	0.015
cellular component	extracellular region part	0.015
molecular function	acetyl-CoA C-acyltransferase activity	0.016
molecular function	misfolded protein binding	0.016
biological process	response to carbohydrate stimulus	0.018
biological process	response to monosaccharide stimulus	0.018

biological process	cellular response to peptide hormone stimulus	0.018
molecular function	carbon-carbon lyase activity	0.02
biological process	cellular response to organic substance	0.02
biological process	response to glucose stimulus	0.021
biological process	negative regulation of carbohydrate metabolic process	0.021
biological process	acetyl-CoA metabolic process	0.022
cellular component	envelope	0.023
molecular function	transferase activity, transferring acyl groups other than amino-acyl groups	0.024
molecular function	small molecule binding	0.024
molecular function	transferase activity	0.028
biological process	regulation of gluconeogenesis	0.029
biological process	cellular biogenic amine metabolic process	0.033
biological process	vitamin metabolic process	0.033
cellular component	intracellular organelle	0.036
molecular function	ammonia-lyase activity	0.038
molecular function	carboxylesterase activity	0.04
biological process	negative regulation of lipid biosynthetic process	0.041
cellular component	organelle	0.041
molecular function	oxidoreductase activity, acting on paired donors, with incorporation or reduction of molecular oxygen, NADH or NADPH as one donor, and incorporation of one atom of oxygen	0.042
biological process	positive regulation of fatty acid metabolic process	0.042
biological process	phosphatidylcholine metabolic process	0.042
biological process	negative regulation of gluconeogenesis	0.046
biological process	positive regulation of fatty acid oxidation	0.046
Meat vs. Tuber (Raw)		
molecular function	catalytic activity	<0.001
molecular function	glutathione transferase activity	<0.001
molecular function	monooxygenase activity	<0.001
molecular function	iron ion binding	<0.001
molecular function	electron carrier activity	<0.001
molecular function	oxidoreductase activity	<0.001
molecular function	oxidoreductase activity, acting on paired donors, with incorporation or reduction of molecular oxygen	<0.001
molecular function	heme binding	<0.001

molecular function	tetrapyrrole binding	<0.001
molecular function	methyl indole-3-acetate esterase activity	<0.001
molecular function	methyl salicylate esterase activity	<0.001
molecular function	methyl jasmonate esterase activity	<0.001
biological process	organic acid metabolic process	<0.001
biological process	cellular amino acid metabolic process	<0.001
biological process	lipid metabolic process	<0.001
biological process	fatty acid metabolic process	<0.001
biological process	organic acid catabolic process	<0.001
biological process	carboxylic acid metabolic process	<0.001
biological process	monocarboxylic acid metabolic process	<0.001
biological process	cellular ketone metabolic process	<0.001
biological process	oxoacid metabolic process	<0.001
biological process	cellular lipid metabolic process	<0.001
biological process	small molecule metabolic process	<0.001
biological process	small molecule catabolic process	<0.001
biological process	carboxylic acid catabolic process	<0.001
biological process	oxidation-reduction process	<0.001
cellular component	cytoplasm	<0.001
cellular component	peroxisome	<0.001
cellular component	endoplasmic reticulum	<0.001
cellular component	microbody	<0.001
cellular component	endoplasmic reticulum part	<0.001
cellular component	cytoplasmic part	<0.001
molecular function	carboxylesterase activity	0.001
molecular function	cofactor binding	0.001
biological process	lipid catabolic process	0.001
biological process	cellular lipid catabolic process	0.001
biological process	cofactor metabolic process	0.001
biological process	monocarboxylic acid catabolic process	0.001
cellular component	endoplasmic reticulum membrane	0.001
biological process	metabolic process	0.002
biological process	response to organic substance	0.002
cellular component	membrane attack complex	0.002
cellular component	nuclear outer membrane-endoplasmic reticulum membrane network	0.002
biological process	acyl-CoA metabolic process	0.003
biological process	coenzyme metabolic process	0.003
biological process	fatty acid catabolic process	0.003
biological process	lipid modification	0.003

biological process	thioester metabolic process	0.003
cellular component	microsome	0.005
molecular function	alkane 1-monooxygenase activity	0.006
biological process	response to chemical stimulus	0.006
cellular component	cell fraction	0.006
cellular component	mitochondrial inner membrane	0.006
cellular component	vesicular fraction	0.006
biological process	response to stress	0.008
biological process	fatty acid beta-oxidation	0.009
molecular function	vitamin binding	0.01
biological process	response to stilbenoid	0.012
molecular function	pyridoxal phosphate binding	0.014
molecular function	vitamin B6 binding	0.014
biological process	fatty acid oxidation	0.014
cellular component	endoplasmic reticulum lumen	0.016
cellular component	organelle inner membrane	0.016
biological process	saturated monocarboxylic acid metabolic process	0.022
biological process	unsaturated monocarboxylic acid metabolic process	0.022
cellular component	cytosol	0.022
biological process	lipid oxidation	0.025
molecular function	carboxylic acid binding	0.027
molecular function	retinyl-palmitate esterase activity	0.033
biological process	cellular modified amino acid metabolic process	0.036
biological process	response to other organism	0.042
biological process	response to biotic stimulus	0.044
Cooked vs. Raw (Meat and Tuber combined)		
biological process	organic acid metabolic process	0.009
biological process	carboxylic acid metabolic process	0.009
biological process	response to chemical stimulus	0.009
biological process	oxoacid metabolic process	0.009
biological process	cellular ketone metabolic process	0.017
biological process	positive regulation of fatty acid oxidation	0.032
molecular function	carbonate dehydratase activity	0.045
Cooked vs. Raw (Tuber)		
molecular function	structural constituent of ribosome	<0.001

molecular function	catalytic activity	<0.001
molecular function	monooxygenase activity	<0.001
molecular function	stearoyl-CoA 9-desaturase activity	<0.001
molecular function	insulin-activated receptor activity	<0.001
molecular function	iron ion binding	<0.001
molecular function	odorant binding	<0.001
molecular function	pheromone binding	<0.001
molecular function	electron carrier activity	<0.001
molecular function	acyl-CoA desaturase activity	<0.001
molecular function	oxidoreductase activity	<0.001
molecular function	oxidoreductase activity, acting on CH-OH group of donors	<0.001
molecular function	oxidoreductase activity, acting on the CH-OH group of donors, NAD or NADP as acceptor	<0.001
molecular function	oxidoreductase activity, acting on paired donors, with incorporation or reduction of molecular oxygen	<0.001
molecular function	oxidoreductase activity, acting on paired donors, with incorporation or reduction of molecular oxygen, reduced flavin or flavoprotein as one donor, and incorporation of one atom of oxygen	<0.001
molecular function	heme binding	<0.001
molecular function	tetrapyrrole binding	<0.001
molecular function	cofactor binding	<0.001
molecular function	coenzyme binding	<0.001
biological process	negative regulation of peptide secretion	<0.001
biological process	organic acid metabolic process	<0.001
biological process	lipid metabolic process	<0.001
biological process	fatty acid metabolic process	<0.001
biological process	insulin receptor signaling pathway	<0.001
biological process	lipid biosynthetic process	<0.001
biological process	response to hexose stimulus	<0.001
biological process	response to glucose stimulus	<0.001
biological process	response to organic substance	<0.001
biological process	regulation of cellular ketone metabolic process	<0.001
biological process	positive regulation of cellular carbohydrate metabolic process	<0.001
biological process	negative regulation of cellular carbohydrate metabolic process	<0.001
biological process	negative regulation of lipid storage	<0.001

biological process	positive regulation of glucose metabolic process	<0.001
biological process	regulation of lipid metabolic process	<0.001
biological process	carboxylic acid metabolic process	<0.001
biological process	monocarboxylic acid metabolic process	<0.001
biological process	cellular response to insulin stimulus	<0.001
biological process	response to monosaccharide stimulus	<0.001
biological process	insulin secretion involved in cellular response to glucose stimulus	<0.001
biological process	cellular ketone metabolic process	<0.001
biological process	response to chemical stimulus	<0.001
biological process	oxoacid metabolic process	<0.001
biological process	cellular lipid metabolic process	<0.001
biological process	small molecule metabolic process	<0.001
biological process	small molecule biosynthetic process	<0.001
biological process	locomotor rhythm	<0.001
biological process	negative regulation of gluconeogenesis	<0.001
biological process	positive regulation of lipid metabolic process	<0.001
biological process	negative regulation of carbohydrate metabolic process	<0.001
biological process	positive regulation of carbohydrate metabolic process	<0.001
biological process	negative regulation of insulin secretion	<0.001
biological process	oxidation-reduction process	<0.001
biological process	regulation of insulin secretion involved in cellular response to glucose stimulus	<0.001
biological process	negative regulation of insulin secretion involved in cellular response to glucose stimulus	<0.001
biological process	mitochondrion morphogenesis	<0.001
biological process	cellular response to chemical stimulus	<0.001
biological process	cellular response to monosaccharide stimulus	<0.001
biological process	cellular response to hexose stimulus	<0.001
biological process	cellular response to glucose stimulus	<0.001
biological process	negative regulation of peptide hormone secretion	<0.001
cellular component	cell fraction	<0.001
cellular component	extracellular region	<0.001
cellular component	membrane attack complex	<0.001
cellular component	membrane fraction	<0.001
cellular component	insoluble fraction	<0.001
cellular component	cytoplasm	<0.001
cellular component	endoplasmic reticulum	<0.001

cellular component	endoplasmic reticulum membrane	<0.001
cellular component	microsome	<0.001
cellular component	cytosol	<0.001
cellular component	ribosome	<0.001
cellular component	small ribosomal subunit	<0.001
cellular component	cytosolic small ribosomal subunit	<0.001
cellular component	vesicular fraction	<0.001
cellular component	ribosomal subunit	<0.001
cellular component	endoplasmic reticulum part	<0.001
cellular component	cytoplasmic part	<0.001
molecular function	transporter activity	0.001
biological process	cellular glucose homeostasis	0.001
biological process	regulation of gluconeogenesis	0.001
biological process	transmembrane receptor protein tyrosine kinase signaling pathway	0.001
biological process	steroid metabolic process	0.001
biological process	negative regulation of hormone secretion	0.001
biological process	cellular response to carbohydrate stimulus	0.001
biological process	cellular response to peptide hormone stimulus	0.001
cellular component	hemoglobin complex	0.001
cellular component	cytosolic ribosome	0.001
cellular component	nuclear outer membrane-endoplasmic reticulum membrane network	0.001
cellular component	cytosolic part	0.001
biological process	regulation of insulin secretion	0.002
biological process	cellular response to lipid	0.002
biological process	regulation of hormone levels	0.003
biological process	response to hormone stimulus	0.004
biological process	regulation of lipid biosynthetic process	0.004
biological process	insulin secretion	0.005
biological process	response to stilbenoid	0.005
biological process	response to carbohydrate stimulus	0.007
biological process	heat generation	0.007
biological process	regulation of carbohydrate biosynthetic process	0.007
biological process	positive regulation of protein kinase B signaling cascade	0.007
biological process	regulation of peptide hormone secretion	0.007
molecular function	structural molecule activity	0.008

molecular function	oxidoreductase activity, acting on paired donors, with oxidation of a pair of donors resulting in the reduction of molecular oxygen to two molecules of water	0.009
biological process	regulation of peptide secretion	0.01
biological process	regulation of peptide transport	0.01
biological process	circadian behavior	0.011
biological process	aerobic respiration	0.013
biological process	response to endogenous stimulus	0.013
cellular component	organelle inner membrane	0.017
biological process	organic acid biosynthetic process	0.018
biological process	carboxylic acid biosynthetic process	0.018
biological process	rhythmic behavior	0.019
biological process	regulation of lipid storage	0.019
biological process	peptide transport	0.019
molecular function	oxygen binding	0.02
biological process	regulation of cellular carbohydrate metabolic process	0.022
cellular component	mitochondrial inner membrane	0.024
biological process	enzyme linked receptor protein signaling pathway	0.027
biological process	peptide hormone secretion	0.028
biological process	cellular response to hormone stimulus	0.028
biological process	cellular response to organic substance	0.028
biological process	negative regulation of lipid metabolic process	0.029
biological process	regulation of carbohydrate metabolic process	0.031
biological process	negative regulation of lipid biosynthetic process	0.031
molecular function	N-acyltransferase activity	0.032
biological process	peptide secretion	0.032
molecular function	oxygen transporter activity	0.034
biological process	cellular respiration	0.035
molecular function	aromatase activity	0.043
biological process	gastrulation with mouth forming second	0.046
biological process	regulation of glucose metabolic process	0.046
biological process	response to insulin stimulus	0.046
biological process	negative regulation of secretion	0.046
cellular component	90S preribosome	0.047
biological process	response to lipid	0.049
Cooked vs. Raw (Meat)		

molecular function	receptor activity	<0.001
biological process	cell activation	<0.001
biological process	type III hypersensitivity	<0.001
biological process	regulation of type III hypersensitivity	<0.001
biological process	positive regulation of type III hypersensitivity	<0.001
biological process	adaptive immune response	<0.001
biological process	immune effector process	<0.001
biological process	myeloid leukocyte activation	<0.001
biological process	neutrophil activation involved in immune response	<0.001
biological process	immune system process	<0.001
biological process	leukocyte mediated immunity	<0.001
biological process	myeloid leukocyte mediated immunity	<0.001
biological process	antigen processing and presentation of peptide antigen via MHC class I	<0.001
biological process	antigen processing and presentation of exogenous peptide antigen	<0.001
biological process	antigen processing and presentation of peptide antigen via MHC class II	<0.001
biological process	antigen processing and presentation of peptide or polysaccharide antigen via MHC class II	<0.001
biological process	positive regulation of acute inflammatory response	<0.001
biological process	regulation of immune system process	<0.001
biological process	positive regulation of immune system process	<0.001
biological process	positive regulation of leukocyte activation	<0.001
biological process	regulation of myeloid leukocyte mediated immunity	<0.001
biological process	defense response	<0.001
biological process	inflammatory response	<0.001
biological process	immune response	<0.001
biological process	response to wounding	<0.001
biological process	antigen processing and presentation	<0.001
biological process	antigen processing and presentation of exogenous antigen	<0.001
biological process	antigen processing and presentation of exogenous peptide antigen via MHC class II	<0.001
biological process	neutrophil chemotaxis	<0.001
biological process	T cell activation	<0.001
biological process	defense response to bacterium	<0.001
biological process	innate immune response	<0.001

biological process	leukocyte activation	<0.001
biological process	lymphocyte activation	<0.001
biological process	antigen processing and presentation of peptide antigen	<0.001
biological process	regulation of phagocytosis	<0.001
biological process	regulation of immune response	<0.001
biological process	response to stimulus	<0.001
cellular component	plasma membrane	<0.001
cellular component	external side of plasma membrane	<0.001
cellular component	cell surface	<0.001
cellular component	membrane	<0.001
cellular component	integral to membrane	<0.001
cellular component	intrinsic to membrane	<0.001
cellular component	MHC protein complex	<0.001
cellular component	MHC class II protein complex	<0.001
cellular component	membrane part	<0.001
cellular component	plasma membrane part	<0.001
cellular component	cell periphery	<0.001
molecular function	immunoglobulin receptor activity	0.001
molecular function	cytokine binding	0.001
biological process	phagocytosis	0.001
biological process	mononuclear cell proliferation	0.001
biological process	T cell proliferation	0.001
biological process	positive regulation of T cell differentiation	0.001
biological process	lymphocyte proliferation	0.001
biological process	positive regulation of cell activation	0.001
molecular function	IgG binding	0.002
biological process	type IIa hypersensitivity	0.002
biological process	regulation of type IIa hypersensitivity	0.002
biological process	positive regulation of type IIa hypersensitivity	0.002
biological process	type II hypersensitivity	0.002
biological process	leukocyte differentiation	0.002
biological process	acute inflammatory response	0.002
biological process	positive regulation of immune effector process	0.002
biological process	positive regulation of adaptive immune response	0.002
biological process	positive regulation of myeloid leukocyte mediated immunity	0.002
biological process	regulation of type II hypersensitivity	0.002
biological process	positive regulation of type II hypersensitivity	0.002
biological process	Fc receptor signaling pathway	0.002

biological process	neutrophil activation	0.002
biological process	positive regulation of lymphocyte differentiation	0.002
biological process	positive regulation of phagocytosis	0.002
biological process	leukocyte migration	0.002
biological process	response to other organism	0.002
biological process	leukocyte proliferation	0.002
molecular function	signal transducer activity	0.003
molecular function	molecular transducer activity	0.003
biological process	regulation of leukocyte activation	0.003
biological process	mast cell activation	0.003
biological process	cytokine production	0.004
biological process	regulation of acute inflammatory response	0.004
biological process	integrin-mediated signaling pathway	0.004
biological process	antigen processing and presentation of exogenous peptide antigen via MHC class I	0.004
biological process	positive regulation of T cell activation	0.004
biological process	phagocytosis, engulfment	0.005
biological process	response to biotic stimulus	0.005
biological process	regulation of endocytosis	0.005
biological process	regulation of T cell differentiation	0.005
biological process	regulation of T cell activation	0.005
biological process	regulation of cell activation	0.005
molecular function	transmembrane signaling receptor activity	0.007
molecular function	signaling receptor activity	0.007
biological process	myeloid cell activation involved in immune response	0.007
biological process	positive regulation of hypersensitivity	0.007
biological process	positive regulation of response to external stimulus	0.007
molecular function	immunoglobulin binding	0.008
biological process	membrane invagination	0.008
biological process	positive regulation of defense response	0.008
biological process	positive regulation of immune response	0.008
biological process	regulation of cytokine production	0.01
biological process	serotonin secretion	0.01
biological process	positive regulation of acute inflammatory response to antigenic stimulus	0.01
biological process	alpha-beta T cell activation	0.011

biological process	positive regulation of adaptive immune response based on somatic recombination of immune receptors built from immunoglobulin superfamily domains	0.012
biological process	regulation of defense response	0.012
biological process	positive regulation of inflammatory response	0.012
biological process	positive regulation of mast cell activation involved in immune response	0.016
biological process	positive regulation of mast cell degranulation	0.016
biological process	lymphocyte mediated immunity	0.017
biological process	response to bacterium	0.017
biological process	regulation of adaptive immune response	0.02
biological process	response to stress	0.02
biological process	leukocyte chemotaxis	0.02
biological process	regulation of lymphocyte differentiation	0.02
biological process	positive regulation of transport	0.02
biological process	regulation of leukocyte mediated immunity	0.023
biological process	regulation of hypersensitivity	0.023
biological process	positive regulation of leukocyte degranulation	0.023
biological process	positive regulation of lymphocyte activation	0.023
molecular function	IgG receptor activity	0.026
molecular function	Rac GTPase activator activity	0.026
biological process	regulation of homeostatic process	0.026
biological process	positive regulation of alpha-beta T cell differentiation	0.027
biological process	cell killing	0.031
biological process	hypersensitivity	0.031
biological process	regulation of acute inflammatory response to antigenic stimulus	0.031
biological process	T cell differentiation	0.031
biological process	positive regulation of mast cell activation	0.031
biological process	positive regulation of B cell differentiation	0.031
cellular component	receptor complex	0.032
molecular function	protein binding	0.033
biological process	adaptive immune response based on somatic recombination of immune receptors built from immunoglobulin superfamily domains	0.035
molecular function	protein complex binding	0.036
biological process	positive regulation of inflammatory response to antigenic stimulus	0.037

biological process	hemopoietic or lymphoid organ development	0.038
biological process	regulation of lymphocyte activation	0.038

Table 0.1: Table showing the enrichment of differentially expressed genes between food substrates and food preparations in the gene ontology (GO). The significant categories their domain and the corresponding FWER (< 0.05) are displayed

KEGG pathway	p value
Meat vs. Tuber (Cooked and Raw combined)	
Metabolic pathways	1.3E-13
Ribosome	1.6E-09
Fatty acid metabolism	9.6E-09
Tryptophan metabolism	8.6E-07
Alanine, aspartate and glutamate metabolism	1.1E-06
Biosynthesis of unsaturated fatty acids	1.7E-06
Arginine and proline metabolism	2.4E-06
Valine, leucine and isoleucine degradation	3.4E-06
PPAR signaling pathway	5E-06
Steroid biosynthesis	1.6E-05
Glycine, serine and threonine metabolism	5E-05
Pyruvate metabolism	0.00021
Drug metabolism - cytochrome P450	0.00042
Fatty acid elongation in mitochondria	0.00051
Synthesis and degradation of ketone bodies	0.00051
Peroxisome	0.00056
Terpenoid backbone biosynthesis	0.00083
Butanoate metabolism	0.00084
Glycolysis / Gluconeogenesis	0.0013
Nitrogen metabolism	0.0028
Metabolism of xenobiotics by cytochrome P450	0.0039
Propanoate metabolism	0.0048
Lysine biosynthesis	0.005
Caffeine metabolism	0.0052
Bile secretion	0.0054
Retinol metabolism	0.006
Lysine degradation	0.0061
Phenylalanine metabolism	0.0063
Histidine metabolism	0.0079
beta-Alanine metabolism	0.01
Glutathione metabolism	0.011
Systemic lupus erythematosus	0.015
Arachidonic acid metabolism	0.021
Glycerolipid metabolism	0.022
Primary bile acid biosynthesis	0.031
Antigen processing and presentation	0.039
Cysteine and methionine metabolism	0.041

Renin-angiotensin system	0.043
Meat vs. Tuber (Cooked)	
Metabolic pathways	6.6E-21
Fatty acid metabolism	2.2E-09
PPAR signaling pathway	2.2E-09
Steroid biosynthesis	5E-08
Valine, leucine and isoleucine degradation	2.2E-07
Alanine, aspartate and glutamate metabolism	8.6E-07
Biosynthesis of unsaturated fatty acids	1.1E-06
Arginine and proline metabolism	1.7E-06
Tryptophan metabolism	1.2E-05
Terpenoid backbone biosynthesis	1.4E-05
Synthesis and degradation of ketone bodies	2E-05
Butanoate metabolism	2.1E-05
Systemic lupus erythematosus	3E-05
Peroxisome	5.7E-05
Glutathione metabolism	8.4E-05
Nitrogen metabolism	0.00018
Pyruvate metabolism	0.00037
Drug metabolism - cytochrome P450	0.00057
Bile secretion	0.00091
Retinol metabolism	0.002
Histidine metabolism	0.0025
Glycolysis / Gluconeogenesis	0.0038
Glycine, serine and threonine metabolism	0.0043
Phenylalanine metabolism	0.0056
Arachidonic acid metabolism	0.0058
Propanoate metabolism	0.01
Glycerolipid metabolism	0.014
Metabolism of xenobiotics by cytochrome P450	0.016
Phenylalanine, tyrosine and tryptophan biosynthesis	0.024
Lysine biosynthesis	0.027
Lysine degradation	0.027
Caffeine metabolism	0.036
Tyrosine metabolism	0.043
Meat vs. Tuber (Raw)	
Retinol metabolism	7E-06
Fatty acid metabolism	1.9E-05
PPAR signaling pathway	3.5E-05

Metabolic pathways	5.1E-05
Synthesis and degradation of ketone bodies	8.3E-05
Ribosome	0.00014
Drug metabolism - cytochrome P450	0.00069
Metabolism of xenobiotics by cytochrome P450	0.0011
Butanoate metabolism	0.0019
Arachidonic acid metabolism	0.0019
Alanine, aspartate and glutamate metabolism	0.0048
Prion diseases	0.0055
Biosynthesis of unsaturated fatty acids	0.0095
Arginine and proline metabolism	0.0097
Peroxisome	0.019
Phenylalanine, tyrosine and tryptophan biosynthesis	0.022
Phenylalanine metabolism	0.022
Valine, leucine and isoleucine degradation	0.023
Glycine, serine and threonine metabolism	0.027
Fat digestion and absorption	0.036
Nitrogen metabolism	0.042
Cooked vs. Raw (Meat and Tuber combined)	
Nitrogen metabolism	0.0009
Osteoclast differentiation	0.0029
Leukocyte transendothelial migration	0.017
Biosynthesis of unsaturated fatty acids	0.019
Adipocytokine signaling pathway	0.019
PPAR signaling pathway	0.024
African trypanosomiasis	0.026
Pentose and glucuronate interconversions	0.028
Toll-like receptor signaling pathway	0.035
Carbohydrate digestion and absorption	0.041
Aldosterone-regulated sodium reabsorption	0.047
Pyruvate metabolism	0.047
Chagas disease (American trypanosomiasis)	0.049
Phagosome	0.049
Cyanoamino acid metabolism	0.05
Cooked vs. Raw (Tuber)	
Retinol metabolism	3E-06
Drug metabolism - cytochrome P450	1.3E-05
PPAR signaling pathway	1.8E-05
Metabolism of xenobiotics by cytochrome P450	2.6E-05

Ribosome	4.1E-05
Pentose and glucuronate interconversions	0.00012
Steroid hormone biosynthesis	0.00031
Phenylalanine, tyrosine and tryptophan biosynthesis	0.00056
Linoleic acid metabolism	0.00068
Tryptophan metabolism	0.00099
Arachidonic acid metabolism	0.0013
Drug metabolism - other enzymes	0.0034
Ascorbate and aldarate metabolism	0.0036
Biosynthesis of unsaturated fatty acids	0.0036
Pyruvate metabolism	0.0039
Butanoate metabolism	0.0049
Metabolic pathways	0.0055
Primary bile acid biosynthesis	0.0062
Phenylalanine metabolism	0.011
Prion diseases	0.011
Nitrogen metabolism	0.021
Fatty acid metabolism	0.031
African trypanosomiasis	0.042
Complement and coagulation cascades	0.047
Amoebiasis	0.047
Cooked vs. Raw (Meat)	
Osteoclast differentiation	4E-14
Staphylococcus aureus infection	2.6E-12
Phagosome	1.1E-10
Leishmaniasis	1.7E-08
Autoimmune thyroid disease	3.3E-08
Antigen processing and presentation	3.9E-08
Cell adhesion molecules (CAMs)	2.1E-07
Allograft rejection	3.5E-07
Graft-versus-host disease	4.9E-07
Asthma	5.2E-07
Type I diabetes mellitus	8.8E-07
Natural killer cell mediated cytotoxicity	1.8E-06
Viral myocarditis	2.3E-06
Systemic lupus erythematosus	8E-06
Intestinal immune network for IgA production	8.1E-05
Chemokine signaling pathway	0.00011
Rheumatoid arthritis	0.00013

Toxoplasmosis	0.00032
Fc gamma R-mediated phagocytosis	0.0004
Hematopoietic cell lineage	0.0015
Endocytosis	0.0019
Chagas disease (American trypanosomiasis)	0.0039
Fc epsilon RI signaling pathway	0.0072
Cytokine-cytokine receptor interaction	0.0077
Leukocyte transendothelial migration	0.0085
African trypanosomiasis	0.042
Salivary secretion	0.045
Jak-STAT signaling pathway	0.048
B cell receptor signaling pathway	0.048

Table 0.2: Table showing the enrichment of differentially expressed genes between food substrates and food preparations in KEGG pathways. The significant pathways and the corresponding p values (< 0.05) are displayed.
